# EVC protein regulates Sonic hedgehog signaling during human intervertebral disc development and degeneration

**DOI:** 10.1016/j.isci.2025.114290

**Published:** 2025-12-04

**Authors:** Zihan Wu, Lizzy Shaw, Christabel T. Dube, Andra-Maria Ionescu, Tengyang Qiu, Anna L. Tierney, Pauline Baird, Sonal Patel, Leo A.H. Zeef, Lindsay J. Birchall, Rachel E. Jennings, Neil A. Hanley, Richard D. Unwin, Judith A. Hoyland, Stephen M. Richardson

**Affiliations:** 1 Division of Cell Matrix Biology and Regenerative Medicine, School of Biological Sciences, Faculty of Biology, Medicine and Health, University of Manchester, Oxford Road, Manchester, UK; 2 Manchester Cell-Matrix Centre, Division of Cell Matrix Biology and Regenerative Medicine, School of Biological Sciences, Faculty of Biology, Medicine and Health, University of Manchester, Oxford Road, Manchester, UK; 3 Centre for Craniofacial & Regenerative Biology, Faculty of Dentistry, Oral & Craniofacial Sciences, King’s College London, London, UK; 4 Division of Cancer Sciences, School of Medical Sciences, Faculty of Biology, Medicine and Health, University of Manchester, Oxford Road, Manchester, UK; 5 Bioinformatics Core Facility, Faculty of Biology, Medicine & Health, University of Manchester, Oxford Road, Manchester, UK; 6 Division of Diabetes, Endocrinology & Gastroenterology, Faculty of Biology, Medicine & Health, University of Manchester, Oxford Road, Manchester, UK; 7 Endocrinology Department, Manchester University NHS Foundation Trust, Manchester, UK; 8 College of Medicine & Health, University of Birmingham, Birmingham, UK; 9 University Hospitals Birmingham NHS Foundation Trust, Birmingham, UK

**Keywords:** Development, Matrix biology, Cilium biology, Signaling

## Abstract

Notochord-derived cells (NCs) in the developing nucleus pulposus (NP) of the intervertebral disc maintain its hydrated extracellular matrix and their aging-associated loss initiates intervertebral disc degeneration, contributing to back pain. To better understand the molecular regulators of NC function, we profiled the proteome of human fetal NP cells and identified Ellis-van Creveld (EVC) protein as highly enriched in NCs. Using mouse models and CRISPR-engineered human NP cells, we show that EVC facilitates Shh signaling, supports NP cell phenotype, and limits fibrotic matrix changes. Loss of EVC reduced Gli3 processing, impaired Shh pathway activity, and altered extracellular matrix organization, while TGF-β signaling suppressed EVC expression indicating crosstalk between these pathways. These findings establish EVC as a key modulator of developmental and homeostatic signaling in the disc and suggest potential therapeutic targets for disc degeneration and fibrosis, providing strategies for preserving NP function and informing regenerative approaches.

## Highlights

• EVC protein is enriched in human fetal notochord-derived cells of the nucleus pulposus
• EVC positively regulates Shh signaling in nucleus pulposus cells
• EVC and Shh pathway maintain NP cell phenotype and reduce fibrosis
• TGF-β signaling downregulates EVC expression

## Graphical abstract

**Figure:**
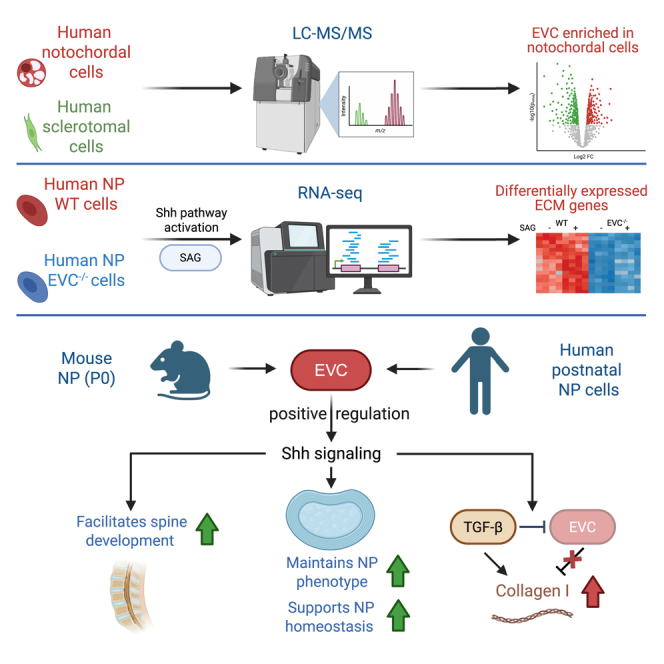

## Introduction

Low back pain is one of the most prevalent musculoskeletal disorders worldwide, imposing significant social and economic burdens.^1^^,^^2^^,^^3^^,^^4^^,^^5^ A primary contributor to this condition is intervertebral disc (IVD) degeneration.^6^^,^^7^ The IVD, a fibrocartilaginous structure situated between vertebral bodies, consists of three main regions: the nucleus pulposus (NP), annulus fibrosus (AF), and cartilaginous endplates (CEPs).^8^^,^^9^ The tissues of the IVD originate from mesoderm-derived notochord and somites. Specifically, the notochord serves as the precursor to the NP, while somites give rise to the vertebral body, AF and CEPs.^7^^,^^10^^,^^11^

In humans, the young healthy NP is populated by abundant notochord-derived cells (NCs), which play a central role in maintaining the IVD’s biomechanical function.^12^^,^^13^ These cells produce a highly hydrated extracellular matrix (ECM) rich in type II collagen and aggrecan (ACAN),^14^^,^^15^ enabling the NP to effectively absorb mechanical loads, an essential characteristic for its shock-absorbing and load-bearing function. However, with skeletal maturation, NCs are gradually lost and replaced by smaller, chondrocyte-like NP cells.^16^^,^^17^ This cellular shift coincides with changes in ECM composition in the NP, including a transition from type II collagen to type I collagen and a reduction in proteoglycan content.^8^^,^^18^^,^^19^ As a result, the ECM becomes more fibrous and less hydrated, disrupting homeostasis and compromising the IVD’s ability to bear mechanical loads.

These changes initiate a degenerative cascade characterized by cell loss, ECM degradation, dehydration, inflammation, and nerve and vascular ingrowth, which are hallmarks of IVD degeneration and sources of chronic pain.^18^^,^^20^^,^^21^ Currently, there is no definitive treatment for low back pain or disc degeneration, and most available therapies focus on relieving symptoms rather than reversing the degenerative process. To develop effective regenerative therapies, a deeper understanding of the cellular and molecular mechanisms underlying IVD development, maintenance, and degeneration is essential.

Evidence suggests that NCs are crucial for IVD homeostasis, as their loss coincides with the onset of degenerative changes, and species that retain NCs into adulthood exhibit delayed disc degeneration.^12^^,^^22^^,^^23^^,^^24^ Within the IVD, NCs display anabolic and protective functions by producing high levels of proteoglycans to enhance ECM hydration and secreting soluble factors that stimulate smaller NP cells to produce a proteoglycan-rich ECM, upregulate anabolic genes, suppress catabolic activity, and inhibit IL-1β-induced cell death.^14^^,^^22^^,^^25^ These functions collectively help the NP to preserve its essential shock-absorbing properties. Consequently, it is hypothesized that NC implantation may halt or even reverse degeneration, offering a potential strategy for relieving low back pain. Little is currently known about the phenotype and regulatory mechanisms of human NCs, and their limited availability poses a challenge for therapeutic applications. Identifying their molecular signatures and regulatory pathways is essential for uncovering factors that may maintain IVD homeostasis and for guiding the differentiation of stem cells into NC-like cells for regenerative therapies.

In previous work, we identified CD24 as a specific cell-surface marker for NCs and used it to isolate and characterize the transcriptome of human fetal NCs. This approach facilitated the identification of novel notochordal markers and enhanced our understanding of NC biology.^26^^,^^27^ Building on these findings, the current study employs CD24-based isolation to analyze the proteomic profile of human fetal NCs. By identifying key proteins and pathways associated with NC function, this study provides new insights into the mechanisms of IVD degeneration and informs the development of regenerative therapeutic strategies.

## Results

### Characterization of proteomic profile of human fetal NCs

**Figure 1:**
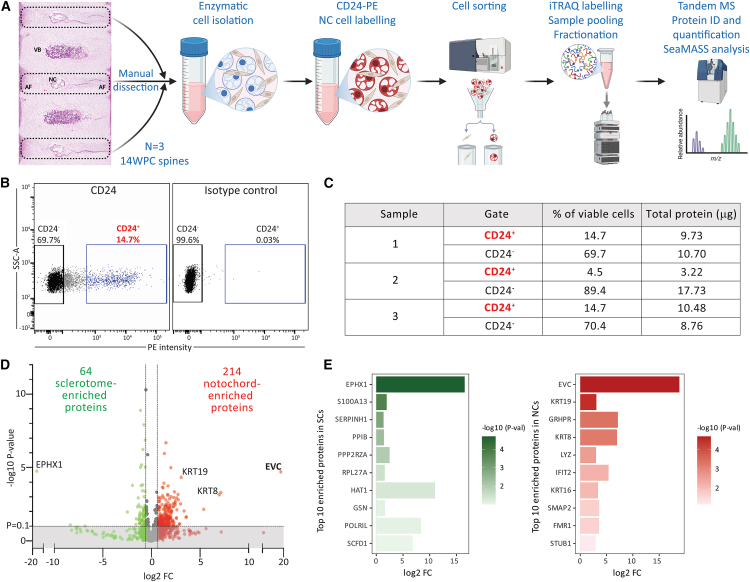
Proteomic profiling of human fetal NCs (A) Schematic of the experimental workflow: IVDs from 14WPC human fetal spines were manually dissected, enzymatically digested, labeled with CD24 antibodies, sorted via FACS, and analyzed using LC-MS/MS with iTRAQ isobaric tags. (B) FACS gating strategy illustrating the separation of CD24^−^ (sclerotomal) and CD24^+^ (notochordal) cell populations. (C) Summary of viable cells proportions (%) and total protein yield from individual samples. (D) Volcano plot displaying DEPs between SCs and NCs. Log2 FC cut off = 1.5; *p*-value cut off = 0.1. (E) Top 10 enriched proteins in sclerotomal (green) and notochordal (red) cells, ranked by *p*-values.

Figure 1A provides an overview of the proteomic workflow used to compare human fetal NCs and sclerotomal cells (SCs). Cell sorting yielded a small CD24^+^ NC population and a larger CD24^−^ SC population (Figure 1B). Viable cell percentages from three independent fetal spine samples and the total protein yield from isolated cells were used for subsequent proteomic analysis (Figure 1C). Principal component analysis (PCA, Figure S1A) and heatmap visualization (Figure S1B) revealed distinct proteomic profiles for CD24^+^ NC and CD24^−^ SC populations. Differential expression analysis identified 214 differentially expressed proteins with increased abundance and 64 proteins with decreased abundance in fetal NCs compared to SCs (Figure 1D). The identified proteins included established notochordal markers (Krt8 and Krt19) along with novel phenotypic markers; the top ten proteins enriched in NC and SC populations are highlighted in Figure 1E.

### EVC is expressed in human NP across lifespan and associates with Shh signaling activity

**Figure 2:**
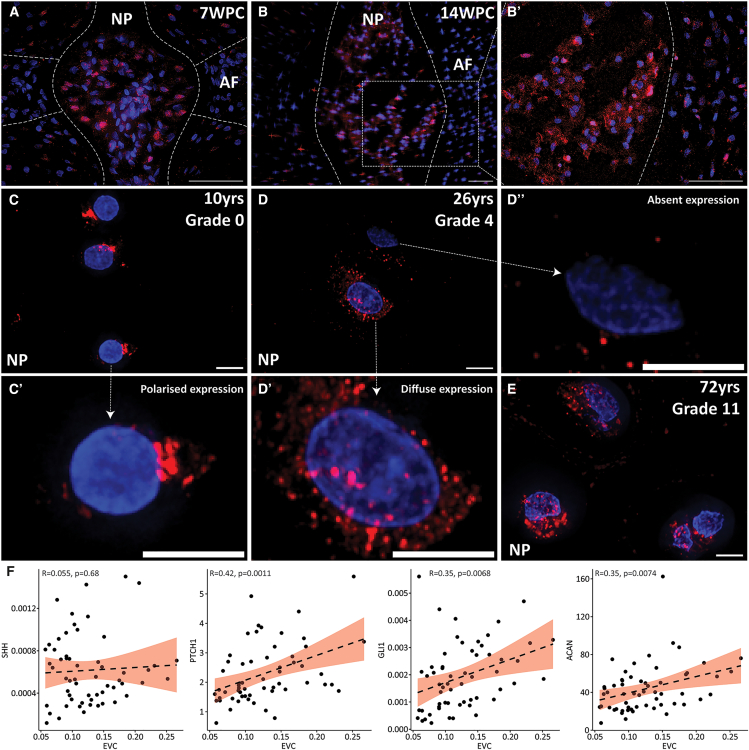
EVC expression in human spine sections and its correlation with Shh signaling and *ACAN* (A–E) IF staining of EVC in human IVDs at various stages: (A) 7WPC, (B) 14WPC IVD with (B′) higher magnification, (C) 10-year-old NP, (D) Grade 4 moderate degenerate NP, and (E) Grade 11 severe degenerate NP. Representative zoomed-in images display distinct EVC expression patterns: (C′) polarized, (D′) diffuse, and (D″) low or absent. Scale bar = 50 μm (A-B′), 5 μm (C-E). (F) Correlation analysis of *EVC* gene expression with *SHH*, *PTCH1*, *GLI1* and *ACAN* across 58 human degenerate NP samples. Relative expression levels (2^-^^ΔCt^) were normalized to the average of *MRPL19* and *GAPDH*. Simple linear regression assessed gene-gene relationships, with R values indicating correlation strength. Each dot represents an individual sample; dotted lines depict predicted trends with 95% confidence intervals. Statistical significance was determined at *p* < 0.05.

Ellis-van Creveld (EVC), identified as the top-enriched protein in the notochord, has not previously been reported in the IVD. To confirm and characterize its expression in human IVD, IF staining was conducted on spine sections across developmental stages. Consistent with the mass spectrometry data, EVC showed substantially stronger staining in the NP region at fetal stages 7WPC (Figure 2A) and 14WPC (Figures 2B and 2B′) compared to surrounding tissues. Expression persisted in pediatric (Figure 2C) and adult (Figures 2D–2E) NP sections, with both single cells and cell clusters displaying immunopositivity. The majority of NP cells were EVC-positive, exhibiting variable patterns including diffuse, polarized, or absent expression. These patterns were observed across all age groups without a clear correlation to developmental stage. EVC is hypothesized to modulate Hedgehog signaling via primary cilia in the IVD. Primary cilia, indicated by Arl13b and γ-Tub staining, were readily detected in NCs at 13WPC (Figures S2A–S2C), but were rarely observed in degenerate NP from older adults (Figures S2D–S2E′).

To examine the role of EVC in Shh signaling, RT-qPCR was conducted on degenerate NP samples from 58 patients aged 40–83 years. Although *SHH* and *EVC* expression did not correlate significantly, *EVC* expression positively correlated with *PTCH1*, *GLI1*, and *ACAN* (Figure 2F). Comparing matched NP and AF samples (*n* = 14; Table S1) revealed no significant difference in *EVC* and *PTCH1* gene expression between NP and AF, but *SHH* and *GLI1* levels were significantly higher in the NP (Figure S3A). This pattern was consistent in comparison between all NP and AF samples (Figure S3B). Additionally, *PTCH1* expression negatively correlated with age in the NP, while other genes showed no significant age association (Figure S3C).

### Evc-/- mice show reduced matrix collagen composition and reduced Hh signaling in the IVD

**Figure 3:**
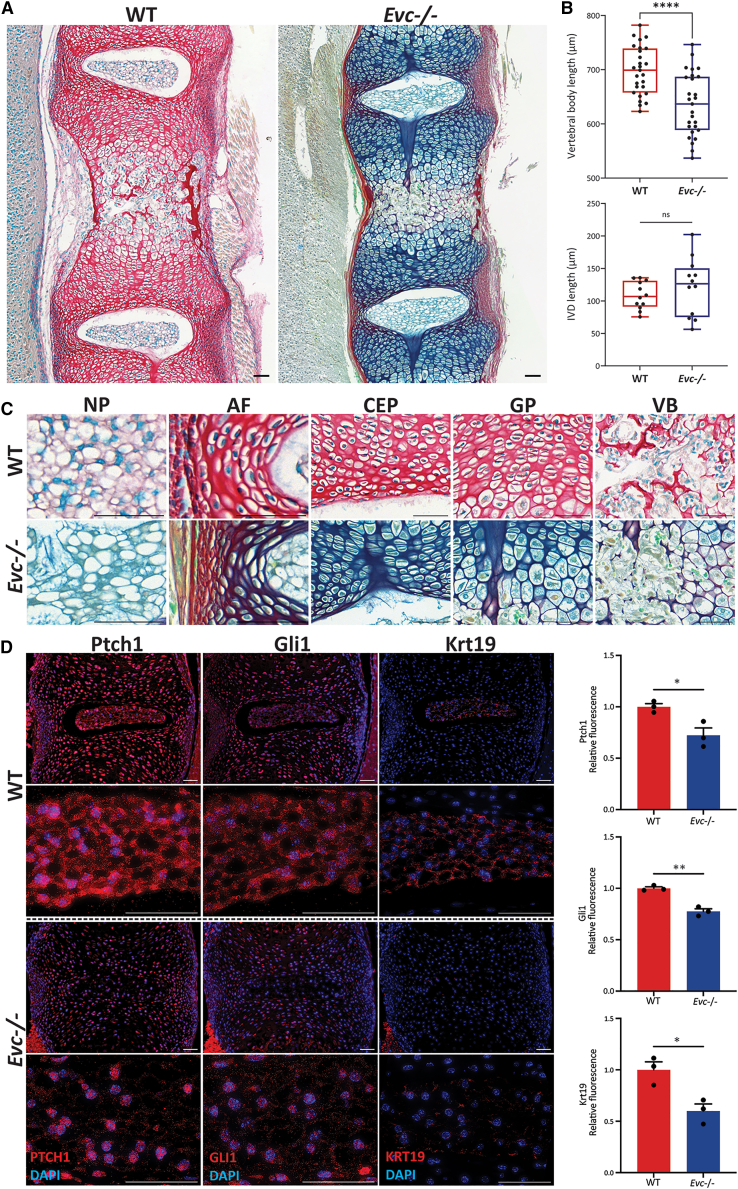
Histomorphometric and molecular analysis of P0 WT and *Evc-/-* mice (A) RGB trichrome staining of IVD sections from WT littermates and *Evc-/-* mice. (B) Quantitative comparison of IVD and vertebral body lengths between WT and *Evc-/-* mice (*n* = 3), presented as mean ± SEM; statistical significance determined by unpaired *t* test. (C) High-magnification images highlighting histological differences in the nucleus pulposus (NP), annulus fibrosus (AF), cartilaginous endplates (CEP), growth plate (GP), and vertebral body (VB). (D) Immunofluorescence staining and quantification of Ptch1, Gli1, and Krt19 expression in IVD sections from WT and *Evc-/-* mice (*n* = 3). Fluorescence intensity normalized to WT averages and presented as mean ± SEM; unpaired *t* test used for statistical analysis. Scale bar = 50 μm for all images. (∗) *p* < 0.05; (∗∗) *p* < 0.01; (∗∗∗∗) *p* < 0.0001.

Phenotypic differences in P0 wild-type (WT) and global *Evc* knockout (*Evc-/-*) mouse spines were assessed using picrosirius red, fast green, and alcian blue (RGB) trichrome staining. WT mice showed strong red collagen staining, while *Evc-/-* mice exhibited reduced red and increased blue/green staining, suggesting lower collagen content in *Evc-/-* spines (Figure 3A). Although IVD length was unaffected, vertebral bodies were significantly shorter in *Evc-/-* mice (Figure 3B). High-magnification images revealed distinct cell and matrix characteristics across spine regions, including NP, AF, CEPs, growth plates, and vertebral bodies (Figure 3C). In the NP, WT cells appeared dark blue with defined cell-matrix boundaries, while *Evc-/-* NP cells were greenish, surrounded by a collagen-reduced matrix. *Evc-/-* mice also showed predominantly reduced collagen in the AF and CEPs. In the growth plate, hypertrophic chondrocytes in *Evc-/-* mice were larger and more rounded. Calcified bone was observed in WT vertebral bodies but was absent in *Evc-/-* mice.

EVC expression was confirmed in P0 WT mouse sections by IF staining, showing presence in both NP and AF regions, high generalized expression in the NP and predominantly cytoplasmic and polarized expression in the AF (Figure S4A). Primary cilia labeling in WT and *Evc-/-* mice revealed association of ciliary markers in both groups, with no observable differences in cilium length (Figure S4B).

IF staining was performed to quantify Shh signaling and assess NP phenotype in P0 *Evc-/-* mice. While Shh expression remained unchanged (Figure S4C), Ptch1 and Gli1 levels were significantly reduced in *Evc-/-* NP, with similar reductions in surrounding tissues. Moreover, the Shh target and NP marker Krt19 was remarkably lower in *Evc-/-* NP (Figure 3D).

### EVC positively regulates Shh signaling and maintains basal Gli3 expression in human NP cells

**Figure 4:**
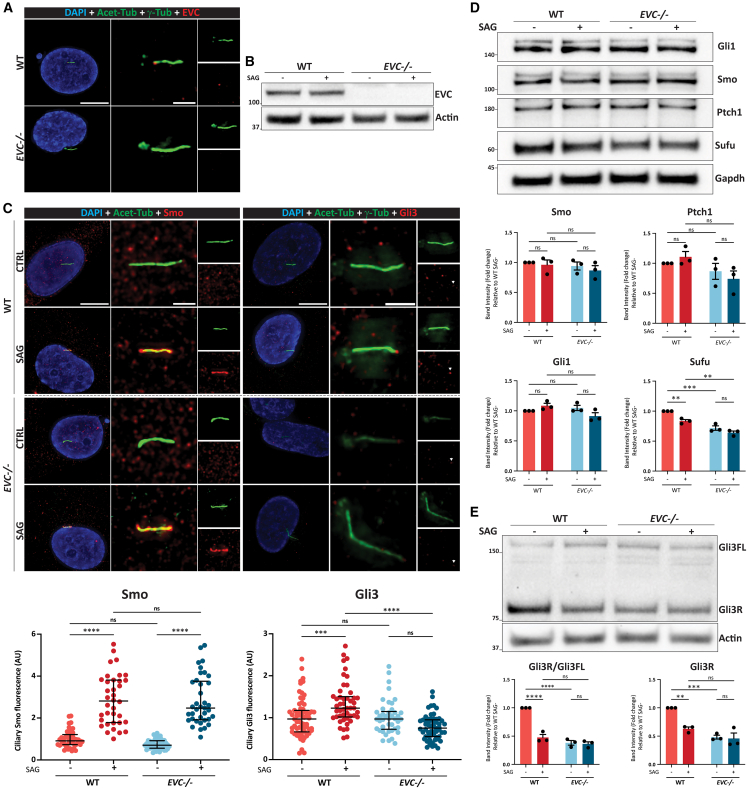
Comparative analysis of WT and *EVC-/-* NP cells (A) Immunocytochemistry illustrating EVC localization in WT and *EVC-/-* NP cells. (B) Western blot analysis of EVC expression in WT and *EVC-/-* cells, with and without SAG treatment. (C) Immunocytochemistry staining and quantification of ciliary Smo and Gli3 in WT and *EVC-/-* cells under basal conditions and following SAG activation (Smo: *n* = 45, 36, 48 and 37 cilia in WT SAG-, WT SAG+, *EVC-/-* SAG-, and *EVC-/-* SAG+, respectively; Gli3: *n* = 67, 48, 42 and 58 cilia in WT SAG-, WT SAG+, *EVC-/-* SAG-, and *EVC-/-* SAG+, respectively). (D–E) Western blot analysis and quantification of Shh pathway components Gli1, Smo, Sufu, Ptch1 and Gli3 in WT and *EVC-/-* cells (*n* = 3), with or without SAG treatment. Data are presented as mean ± SEM. Ciliary fluorescence was normalized to WT SAG- averages. Protein expression was normalized to the corresponding WT SAG- sample. Scale bar = 10 μm in DAPI-stained panels and 2 μm in magnified images. Statistical significance was determined using two-way ANOVA followed by Tukey’s multiple comparison test. (∗∗) *p* < 0.01; (∗∗∗) *p* < 0.001; (∗∗∗∗) *p* < 0.0001.

To elucidate EVC’s role in NP cells, CRISPR knockout of *EVC* was conducted on human NP cell line NP105 using sgRNAs targeting exon 4, followed by expansion of a monoclonal line. Sanger sequencing confirmed a “G” deletion (Figure S5A). ICC staining showed EVC localization at the primary cilium base in WT NP cells, marked by γ-Tub and Acet-Tub, which was absent in *EVC-/-* cells (Figure 4A). Western blot confirmed *EVC* knockout, with EVC expression in WT cells unaffected by smoothened agonist (SAG)-induced Hedgehog pathway activation (Figure 4B). Analysis of primary cilia revealed that while SAG and *EVC* knockout did not impact cilium length, ciliation was slightly reduced in *EVC-/-* cells upon SAG activation (Figure S5B).

To examine Hh pathway activation in NP cells and the effects of *EVC* knockout, ciliary accumulation of Smo and Gli3 was evaluated in WT and *EVC-/-* cells with and without SAG stimulation. Smo accumulated normally in both groups after SAG treatment, with no difference in intensity, but Gli3 enrichment occurred only in WT cells, showing significantly higher levels than in *EVC-/-* cells with SAG (Figure 4C). Western blot analysis showed no differences in Smo, Ptch1, or Gli1 levels across groups, while Sufu downregulated in WT cells upon SAG. Basal Sufu levels were significantly lower in *EVC-/-* cells and remained unchanged after SAG activation (Figure 4D). Gli3 analysis demonstrated that SAG reduced the Gli3R/Gli3FL ratio and Gli3R expression in WT cells, but not in *EVC-/-* cells. Knockout of *EVC* significantly reduced basal Gli3R levels, which were similar to SAG-stimulated levels in WT cells (Figure 4E). RT-qPCR analysis of *GLI3* mRNA revealed that *GLI3* mRNA levels in WT cells significantly declined following exposure to SAG, and the gene expression level of *GLI3* in *EVC-/-* cells was significantly lower than in WT cells and did not change following SAG stimulation (Figure S5C).

### EVC and Shh pathway regulate ECM composition, and EVC deficiency attenuates Shh response in NP cells

**Figure 5:**
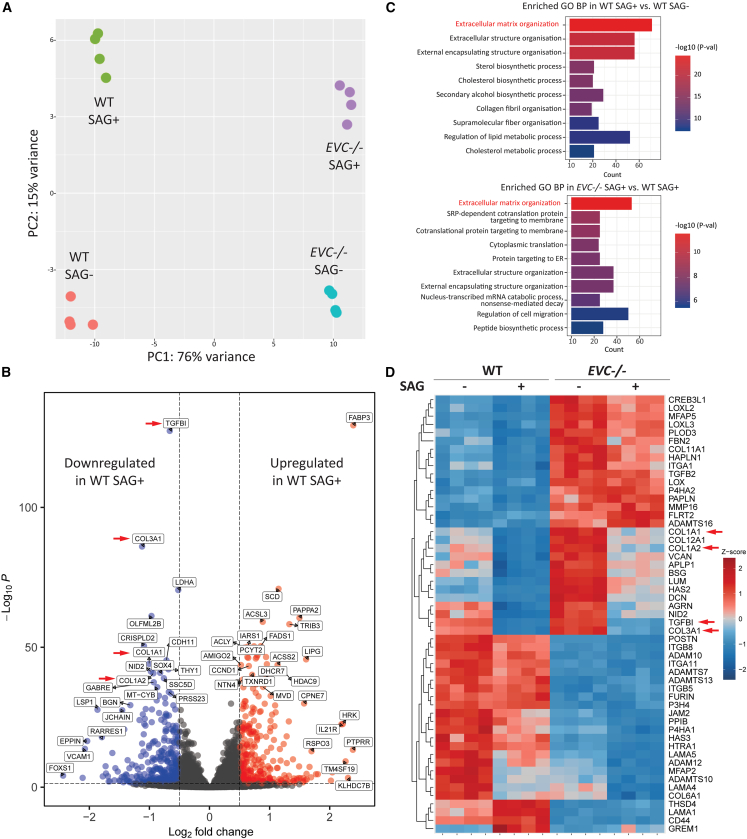
RNA-seq analysis of WT and *EVC-/-* cells with or without SAG treatment (A) PCA plot showing transcriptional differences across experimental groups (*n* = 4 for each group). (B) Volcano plot showing downregulated and upregulated genes in WT NP cells following SAG stimulation. LogFC cut off = 0.5; *p*-value cut off = 0.05. (C) Top 10 enriched GO biological process (BP) terms from WT SAG+ vs. WT SAG- and *EVC-/-* SAG+ vs. WT SAG+ comparisons, ranked by p-adjust values. (D) Heatmap of ECM-related gene expression from the “ECM organization” GO BP term in *EVC-/-* SAG+ vs. WT SAG+ comparison.

To explore the role of Shh and EVC in NP cells, bulk RNA-seq was performed on WT and *EVC-/-* cells in the presence and absence of SAG, with each group containing four replicates. PCA analysis revealed distinct gene expression distributions among the groups (Figure 5A). RNA-seq data for Shh pathway components *SMO*, *PTCH1*, *GLI1*, *GLI3*, and *SUFU* levels were consistent with western blot findings (Figure S6A). Analysis of known Shh targets, including *CCND1*, *BCL2*, and *MYC*, confirmed significant upregulation in WT but not in *EVC-/-* cells following SAG activation (Figure S6B).

Initial differential expression analysis in WT cells revealed that Shh pathway activation resulted in the downregulation of key ECM genes, including *COL1A1*, *COL1A2*, *COL3A1*, and *TGFBI*, which are typically upregulated in association with IVD degeneration (Figure 5B). Volcano plots for additional paired group comparisons illustrated these distribution patterns (Figure S6C). Gene ontology (GO) biological process enrichment analysis highlighted “ECM organization” as a top-ranked term in the comparison between WT control and SAG-treated WT cells, with this term remaining prominent across other comparisons (Figures 5C and S6D).

To identify differential ECM genes between WT and *EVC-/-* cells under SAG treatment, a heatmap of 53 genes in this category was plotted, showing expression levels across all conditions (Figure 5D). Shh activation downregulated *COL1A1*, *COL1A2*, and *COL3A1* in both WT and *EVC-/-* cells. However, baseline expression of these genes was significantly elevated in *EVC-/-* cells compared to WT, and, despite reduction following SAG treatment, *EVC-/-* cells maintained higher expression levels than SAG-treated WT, approximating WT control levels.

### Shh activity and EVC expression reduce NP fibrosis, maintain cell phenotype, and associate with TGF-β signaling

**Figure 6:**
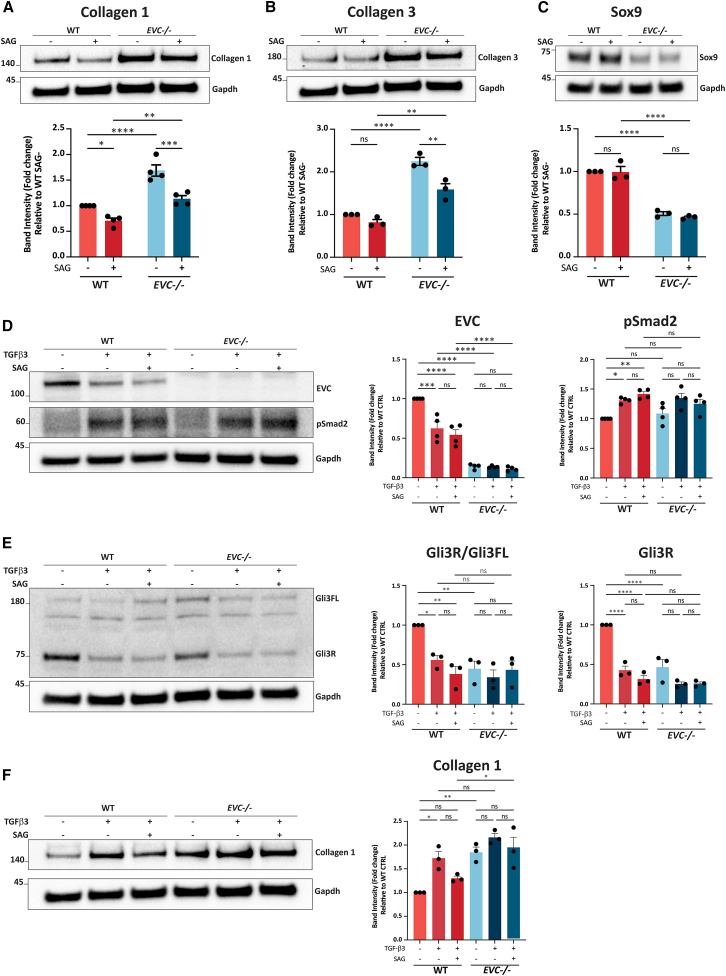
Protein level analysis in WT and *EVC-/-* cells under pathway activation (A–C) Western blot analysis of Collagen 1 (*n* = 4), Collagen 3 (*n* = 3), and Sox9 (*n* = 3) in WT and *EVC-/-* cells with and without SAG treatment. (D–F) Western blot analysis of EVC, pSmad2 (*n* = 4), Gli3, and Collagen 1 (*n* = 3) in WT and *EVC-/-* cells under control, TGF-β3, and combined SAG/TGF-β3 treatments. Protein levels were normalized to WT SAG- samples. Data are presented as mean ± SEM. Statistical significance was determined by two-way ANOVA followed by Tukey’s multiple comparison test. (∗) *p* < 0.05; (∗∗) *p* < 0.01; (∗∗∗) *p* < 0.001; (∗∗∗∗) *p* < 0.0001.

To validate RNA-seq findings, western blot analysis was performed to assess protein levels of collagen 1, collagen 3, and Sox9, a key transcription factor essential for chondrogenic differentiation and a well-established NP phenotype marker due to its critical role in ECM synthesis and NP cell phenotype maintenance. Consistent with RNA-seq data, collagen 1 protein levels were significantly reduced in both WT and *EVC-/-* cells after SAG treatment, with *EVC-/-* cells consistently showing elevated levels compared to WT cells (Figure 6A). For collagen 3, no significant change was observed in WT cells after SAG stimulation. In contrast, *EVC-/-* cells displayed a significant reduction upon SAG activation, yet collagen 3 levels remained significantly higher in both control and SAG-treated conditions compared to WT (Figure 6B). Regarding Sox9, RNA-seq data indicated upregulation in WT cells in response to SAG, whereas *EVC-/-* cells exhibited reduced Sox9 expression that did not respond to SAG stimulation (Figure S7A). However, western blot analysis revealed no significant change in Sox9 protein levels in WT cells following SAG activation (Figure 6C). In *EVC-/-* cells, Sox9 protein expression was significantly lower than in WT and remained unchanged after SAG treatment, aligning with the transcriptomic data. Analysis of collagen 2 expression showed no significant differences across groups in both RNA-seq and Western blot results (Figures S7A–S7B).

Given TGF-β signaling’s role in collagen synthesis in NP cells, further analysis explored potential crosstalk between TGF-β, Shh, and EVC pathways. RNA-seq data indicated altered TGF-β signaling components in response to *EVC* knockout and SAG treatment (Figure S7C). TGF-β3 was used to activate the TGF-β pathway in human WT and EVC-/- cells, revealing minimal impact on Smad2 phosphorylation (pSmad2) levels following TGF-β3 treatment, with EVC expression downregulated by TGF-β activation (Figure 6D). Gli3R/FL ratio and Gli3R expression were significantly reduced after TGF-β3 stimulation in WT cells, which was unaffected by co-stimulation with SAG. These changes were not statistically significant in *EVC-/-* cells (Figure 6E). Collagen 1 analysis showed a significant increase in WT cells after TGF-β3 treatment, with a reduction upon co-stimulation with SAG. *EVC-/-* cells consistently displayed higher collagen 1 levels with no significant changes after TGF-β3 or co-stimulation (Figure 6F).

## Discussion

The proteomic profiling of human NCs in this study revealed both well-established markers (e.g., Krt8 and Krt19) and previously unrecognized proteins that distinguish NCs from surrounding SCs. Notably, EVC was identified for the first time in the human IVD and emerged as the top enriched protein in NCs. *EVC* is a causal gene of EvC syndrome, a rare, recessive congenital skeletal dysplasia characterized by short ribs, short limbs, polydactyly, and dystrophic hair and teeth.^28^^,^^29^^,^^30^ Its high expression in the notochord suggests that EVC may serve as an NC marker at least during the human fetal stage. Moreover, EVC persists throughout NP formation, development, and degeneration, implying a continuous role in IVD homeostasis. Although the SC-abundant proteins identified were not extensively investigated, several candidate markers, particularly EPHX1 and HAT1, underscore the molecular heterogeneity between NCs and SCs.

EVC localizes to the base of primary cilium, a critical sensory organelle that extends from the cell surface to facilitate reception and transduction of extracellular signals. Hh signaling, a well-established cilium-dependent pathway, is positively regulated by EVC in chondrocytes and fibroblasts.^31^^,^^32^^,^^33^^,^^34^^,^^35^ In our study, we detected EVC in various subcellular locations within human NP cells across different developmental stages. Its polarized expression aligns with the ciliary location, supporting the role of EVC in Hh signal transduction. Previous work has shown that mouse IVD cells are ciliated, and defects in primary cilia disrupt cell survival and Hh signaling, impairing IVD cell organization and function. Furthermore, primary cilia in the mouse NP decrease during age-induced IVD degeneration.^36^^,^^37^ Our observations in humans mirror these findings: extensive ciliation was evident in human NCs during early life, whereas ciliated cells were rarely observed in degenerate NP tissue from older adults. These results suggest that ciliary loss may contribute to IVD degeneration and highlight the potential benefits of preserving or restoring ciliary function to maintain NP phenotype and overall disc function.

Ptch1 and Gli1 are key downstream targets of Hh signaling, and their expression markedly decreased in the growth plates of *Evc-/-* mouse embryos. In our study, *EVC* showed a positive correlation with *PTCH1* and *GLI1* expression, supporting its role as a positive modulator of Shh signaling in the NP. In line with previous reports showing that Shh signaling declines with age in the IVD,^38^^,^^39^^,^^40^ a significant negative correlation between *PTCH1* expression and age was observed. However, the correlations involving *SHH* and *GLI1* may have been too late to detect since all samples in our cohort were classified as degenerate. Notably, a positive correlation also emerged between *EVC* and *ACAN*, a major NP proteoglycan essential for ECM hydration and IVD function, suggesting that EVC may contribute to maintaining ECM homeostasis.

*Evc-/-* mouse models recapitulate key features of human EvC syndrome, including short stature and dental anomalies. Skeletal dysplasia in these models results from reduced chondrocyte proliferation and disrupted chondrocyte hypertrophy during long bone development, with affected regions exhibiting markedly diminished Hh signaling.^31^^,^^32^^,^^41^ In our *in vivo Evc* knockout model, *Evc* deficiency led to widespread reductions in spinal collagen deposition, delayed endochondral mineralization, and shortened vertebrae, which mirror the limb and rib shortening observed in EvC syndrome. Overall, *Evc-/-* mice exhibit delayed embryonic spine development.

While high EVC expression was detected in human NP compared to surrounding tissues, this expression pattern was not as obvious in the mouse IVD. This may be attributed to the different developmental stages examined—early fetal development in human IVDs versus post-natal IVD in mice. Additionally, while polarized EVC expression in mouse IVD cells supports its ciliary localization, we also observed notable nuclear expression in mouse NP cells compared to surrounding cells. These findings suggest species-specific expression patterns and distinct functional roles of EVC across cell types and developmental stages. Moreover, our observation also indicates that EVC is not required for ciliogenesis in the mouse NP during IVD formation.

Shh is a major NC marker with essential roles in IVD formation and postnatal maintenance. It is crucial for the transition from notochord to NP during embryonic development,^42^^,^^43^ and Shh secreted by postnatal NP cells maintains the structure and function of both the NP and surrounding tissues. Inhibition of Shh signaling disrupts NP cell organization, proliferation, and the expression of key NP markers including Brachyury, Sox9, Krt19, ACAN, and collagen 2. Shh signaling in the IVD declines with age and its downregulation has been suggested to be associated with IVD degeneration.^38^^,^^39^^,^^40^ We therefore examined Shh and its associated NP markers in the NP. Previous studies have shown that *Evc* knockout does not affect Ihh expression in mouse growth plates.^31^^,^^32^^,^^44^ Consistently, our current study confirms that Shh expression in the mouse NP remains unchanged by *EVC* deficiency, but we observed a significant reduction in Shh signaling activity. This confirms EVC’s role as a positive regulator of the Shh pathway during IVD development. Moreover, *EVC* knockout resulted in an early reduction in Krt19, a well-established NP marker and reported Shh target that normally persists for at least 3–5 months in mice before notably declining by one year. Given its close association with Shh signaling and the link between reduced Krt19 levels and age-related degeneration,^39^^,^^45^^,^^46^ the premature loss of Krt19 in the absence of *EVC* may promote fibrosis, impair disc function, and accelerate IVD degeneration. These findings suggest that EVC may contribute to maintaining NP phenotype and overall disc homeostasis.

Previous studies have employed siRNA knockdown or knockout mouse chondrocytes and fibroblasts to investigate EVC function. In the present study, we established a CRISPR-based *EVC* knockout human NP cell model to examine EVC expression and function in the NP system. We confirmed the presence of EVC in human NP cells, its localization at the ciliary base, and the effects of its knockout. Smo accumulation and activation within the primary cilium are crucial for Hh signaling transduction. Once sufficiently enriched and activated, Smo recruits Gli transcription factors to the ciliary tip, where they become activated and translocate to the nucleus to regulate Hh-responsive gene expression.^47^^,^^48^^,^^49^ Previous studies have shown that EVC enhances signal transduction by forming a complex with Smo at the primary cilium to promote full Smo activation and by indirectly facilitating dissociation of the Sufu/Gli complex to promote Gli activation.^32^^,^^33^^,^^34^^,^^35^^,^^50^ Using SAG to activate the Hh pathway, we found that while EVC is not required for Smo accumulation in NP cells, it is essential for subsequent Gli3 accumulation in cilia. This indicates that EVC in NP cells is required for proper Hh pathway transduction, consistent with previous models. Surprisingly, SAG-induced Shh pathway activation did not upregulate Ptch1 or Gli1 expression in NP cells. However, analysis of Gli3 protein confirmed EVC as a positive regulator of Shh signaling in NP cells. Unlike previous studies, *EVC* knockout in NP cells affected Hh pathway activity even before pathway activation, as evidenced by reduced basal levels of Gli3R and Sufu. Although Gli3 processing involves a complex regulatory network,^51^ this reduction may result from decreased *GLI3* transcription or lower Sufu levels due to *EVC* knockout. Additionally, neither Shh pathway activation nor EVC expression greatly influenced ciliogenesis in human NP cells.

Although EVC is established as a positive regulator of Hh signaling, its precise downstream targets remain incompletely understood. While Shh signaling is known to support NP maintenance, the mechanisms and regulatory targets underlying this function require further elucidation. To address this, we conducted bulk RNA-seq on NP cells subjected to Shh pathway manipulation, with PCA validating distinct and reproducible phenotypes following *EVC* knockout and SAG treatment. Consistent with our earlier findings, RNA-seq revealed no induction of *PTCH1* or *GLI1* after SAG treatment, downregulated *GLI3* and *SUFU* expression upon *EVC* knockout, and reduced or absent Hh target regulation in *EVC-/-* cells, further supporting EVC’s role as a positive regulator of Shh signaling in NP cells. Interestingly, certain Hh targets exhibited elevated basal expression under *EVC* deficiency, possibly due to disrupted Gli3R processing, which suggests a complex interplay between basal Gli3R levels and Gli3FL activation resulting in the phenotypes observed. The absence of PTCH1 and Gli1 induction following SAG treatment can likely be attributed to minimal Gli2 expression in NP cells, as both immunostaining and RNA-seq analyses showed that Gli2 is barely detectable in this context. This agrees with transcriptomic studies reporting reduced Gli2 expression in postnatal mouse NP.^52^ Given that Gli2 is the main effector of Shh signaling activation,^47^ its absence likely limits the canonical Gli2–Gli1/Ptch1 axis, thereby explaining the poor induction of Shh signaling in NP cells. We therefore propose that Gli3 serves as the principal regulator of Shh signaling in NP cells. Furthermore, a recent study reported that genetic inactivation of Gli2 in mouse disc cells does not produce gross NP defects but mainly affects growth plates, whereas Gli3 is more critical for disc cell phenotypes.^53^ Gli3 may be a more suitable target for future NP-focused Hh pathway studies and applications in NP biology. Accordingly, an in-depth exploration of the mechanistic association between EVC and Gli3, including its regulation of Gli3 processing and their specific roles in signaling pathways and cellular phenotypes, will be required.

ECM organization emerged as the top enriched GO term following *EVC* knockout and SAG activation. Several fibrotic markers including *COL1A1*, *COL1A2*, *COL3A1*, and *TGFBI*, often linked to IVD degeneration,^18^^,^^54^ were downregulated upon Shh signaling activation, whereas *EVC* deficiency increased fibrosis-related gene expression and attenuated the downregulatory effects of Shh. Thus, both Shh signaling and EVC appear to play roles in mitigating NP fibrosis. Since Shh signaling declines with aging, the loss of its antifibrotic function may initiate or accelerate disc degeneration. EVC positively modulates Shh signaling and EVC alone is sufficient to inhibit these fibrosis-associated markers, suggesting that potential downregulation or reduced function of EVC could contribute to the degenerative process. Sox9 was upregulated by SAG activation and its expression was sustained by EVC. These findings, further validated at the protein level, reinforce the role of EVC and the Shh pathway in reducing NP fibrosis and maintaining NP phenotype. Although increased Sox9 expression typically correlates with high collagen 2 levels, no corresponding changes were observed, likely due to low collagen 2 expression in our 2D cell culture model. Future studies using 3D biomaterial models or *in vivo* systems would be more appropriate to assess changes in collagen 2 and ACAN. Finally, although EVC is required for full Shh pathway activation, some SAG-induced responses still occurred in *EVC-/-* cells, possibly driven by normal Smo accumulation partially regulating certain downstream effects. Further studies are necessary to clarify these mechanisms.

The TGF-β pathway is another key regulator of IVD development and homeostasis,^55^ as well as a master regulator of fibrosis.^56^^,^^57^ Our findings indicate that *EVC* knockout affects certain TGF-β pathway components, suggesting an interplay between Shh/EVC and TGF-β signaling in NP cells. While SAG activation or *EVC* deficiency had minimal impact on TGF-β signaling, assessed by TGF-β3 treatment and pSmad2 levels, TGF-β activation unexpectedly reduced EVC expression. Given that EVC facilitates Shh signaling transduction, this may allow TGF-β activation to suppress the Shh pathway. These results align with embryological evidence that Shh signaling is gradually repressed by TGF-β as development progresses. In the NP system, this shift is reflected by a decrease in Shh pathway components and a corresponding increase in TGF-β pathway components between E12.5 and P0 in mouse NP.^52^ Notably, this transition coincides with high EVC expression during early development, suggesting that TGF-β may directly target EVC for downregulation and thereby contribute to the suppression of Shh signaling during this transition.

**Figure 7:**
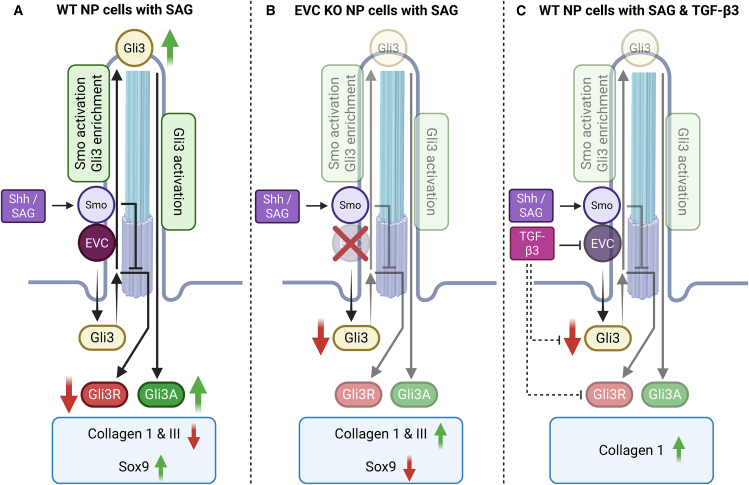
Model of EVC-mediated regulation of Shh and TGF-β signaling in human NP cells (A) EVC promotes Smo activation and Gli3 enrichment at the ciliary tip, facilitating Gli3A formation. It also maintains basal Gli3 expression and Gli3R levels. (B) Loss of EVC impairs Shh signaling activation and reduces Gli3 expression and processing, leading to decreased Sox9 and increased Collagen 1/3 expression. (C) TGF-β signaling decreases EVC levels, thereby attenuating Shh activity and basal Gli3 expression, and may also directly repress Gli3, resulting in increased Collagen 1 expression.

We demonstrated that EVC is crucial for maintaining basal Gli3 expression. The TGF-β-mediated reduction of EVC confirmed that EVC is required for proper Gli3 processing, although TGF-β might also directly modulate Gli3. Extensive research has shown that TGF-β signaling can regulate Gli1 and Gli2 independently of Smo to interact with the Hh pathway.^58^^,^^59^^,^^60^^,^^61^^,^^62^ Here we extend these findings by showing that reductions in both EVC and Gli3 expression following TGF-β activation may alter Shh signaling, with EVC potentially acting as a mediator in the complex crosstalk between them. A schematic summarizing the proposed EVC/Shh-TGF-β interactions is shown in Figure 7.

Collagen 1 is a principal target of TGF-β signaling. Our analysis of collagen 1 expression levels under various conditions again suggests that both Shh and EVC help prevent excessive fibrosis. Indeed, in *EVC*-deficient cells, TGF-β signaling showed limited capacity to further elevate collagen 1 levels. We propose that TGF-β may, at least partially, promote collagen synthesis by downregulating EVC expression, although this hypothesis requires further validation, for example through an *EVC* rescue experiment, which would also help determine whether EVC is sufficient to activate downstream Shh targets. Given the conserved roles of Shh and TGF-β pathways, this regulatory mechanism may extend beyond the NP. Since TGF-β is a central regulator of fibrosis and EVC is expressed in multiple tissues, strategies to enhance EVC expression or function, such as *EVC* overexpression^32^^,^^63^ or modulation of proteins like IQCE and EFCAB7^50^ that stabilize EVC at the cilium, could offer a promising therapeutic approach for managing degenerative and fibrotic diseases.

Overall, our study using both human and mouse models provides novel insights into the expression and localization of EVC, ciliogenesis, and the role of the EVC/Shh signaling pathway in the NP. EVC expression and primary cilia were confirmed in both human and mouse NP cells, and our data indicate that EVC is not required for ciliogenesis. We demonstrated that EVC is a positive regulator of the Shh signaling pathway in both models and plays an important role in maintaining NP phenotype, as shown by its regulation of key markers (Krt19 in mouse NP and Sox9 in human NP cells). Notable differences include higher EVC expression in the human NP relative to adjacent tissues and distinct regulation of fibrotic outcomes. Specifically, in mice, EVC facilitated embryonic spine development by promoting vertebral length, matrix collagen deposition, and vertebral ossification, whereas in human NP cells, EVC and Shh signaling appear to prevent fibrosis by downregulating collagen 1, collagen 3, and *TGFBI*. Importantly, a key point for this study is that the timing and dynamics of NC loss differ markedly between species. In humans, NC cells are lost early in childhood, whereas in mice they are maintained well into adulthood. These differences likely influence steady-state EVC expression and define the cell populations that remain responsive to Shh signaling in the NP, thus contributing to stage- and cell-type-specific roles of EVC and the overall activity and function of the Shh pathway. In addition, diverse EVC expression patterns and potential EVC functions beyond the cilium and Shh pathway, together with differences in ciliary phenotypes and mechanical loading environments between human and mouse IVDs, may further modulate how EVC regulates disc development and maintenance. Taken together, these considerations highlight the need for caution when extrapolating mouse findings to human NP biology and argue that therapeutic strategies that target EVC/Shh should be guided by human tissue and cell data, ideally validated in models that recapitulate human NP composition.

In conclusion, EVC regulates Shh signaling in the NP, supporting development, ECM maintenance, and fibrosis reduction, while mediating crosstalk with TGF-β signaling. Its role in IVD formation and maintenance suggests that EVC may present a potential target for preventing degeneration and promoting disc homeostasis and regeneration. Importantly, beyond the well-recognized role of the Hh pathway in embryonic development, our findings suggest its continued influence on ECM composition during tissue maintenance and identify the responsive molecular targets of the EVC/Shh pathway. The demonstration of EVC-Shh-TGF-β interactions also provides important insights into the complexity of signaling crosstalk. Together with our perspective that ciliary biology contributes to tissue homeostasis and ECM regulation, this study offers broader insights into the role of the ECM in health and disease, with translational significance for degenerative and fibrotic disorders and implications for future regenerative therapies.

### Limitations of the study

Our study has some limitations. Firstly, the use of a global *Evc* knockout mouse model does not allow us to distinguish tissue-specific effects or isolate the impact of reduced Hh activity in adjacent regions. Secondly, our analysis of P0 mice limited our ability to examine age-related changes in EVC expression, localization, and their association with NP phenotype, leaving it unresolved whether EVC contributes to preventing or delaying IVD degeneration over time. Future studies using adult-onset models will be important to determine whether loss of EVC accelerates age-related disc degeneration and fibrosis and whether enhancing or restoring EVC function could maintain or mitigate the degenerative changes. Moreover, employing aged mouse models, rat tail degeneration models, or large-scale human IVD cohorts to correlate EVC expression patterns with pathological features, as well as assessing functional consequences such as locomotor performance, spinal biomechanics, and long-term outcomes, will be important directions for future research. Finally, while our experiments focused on TGF-β signaling via TGF-β3 stimulation and Smad2 phosphorylation, other key mediators such as Smad3, Smad4, and BMP-associated Smads 1/5/9,^64^^,^^65^ may also interact with EVC/Shh and influence ECM regulation and fibrosis. Extending these analyses to an *in vivo* setting, including TGF-β signaling components, fibrosis-associated markers, and pathway crosstalk, represents an important future direction for understanding fibrosis and exploring therapeutic potential. Further studies addressing these factors will provide a more comprehensive view of EVC and its role in signaling interplay.

## Resource availability

### Lead contact

Requests for further information and resources should be directed to and will be fulfilled by the lead contact, Stephen Richardson (s.richardson@manchester.ac.uk).

### Materials availability

This study did not generate new unique reagents.

### Data and code availability

• The proteomic dataset generated and analyzed during the study is provided as supplemental information. The RNA-seq datasets generated and analyzed during the current study are available from Array Express: https://www.ebi.ac.uk/arrayexpress/; accession number E-MTAB-15392.
• This paper does not report original code.
• Any additional information required to reanalyze the data reported in this paper is available from the lead contact upon request (Data S1).

## Acknowledgments

Professor Abigail Tucker (King’s College London) is thanked for provision of *Evc-/-* mice and Professor Andrew W. Dowsey (10.13039/501100000883University of Bristol) is thanked for his support with analysis of the proteomic data. This study was additionally supported by the 10.13039/501100000770University of Manchester Genome Editing Unit, as well as the Biological Mass Spectrometry (BioMS; RRID: SCR_020987), Genomic Technologies, Bioinformatics, Bioimaging, and Flow Cytometry core facilities. The Flow Cytometry Core Facility is supported in part by the 10.13039/501100000770University of Manchester, 10.13039/501100012041Arthritis UK and the Manchester Center for Collaborative Inflammation Research (10.13039/501100022187MCCIR). The Bioimaging Facility microscopes used in this study were purchased with grants from 10.13039/501100000268BBSRC, 10.13039/100004440Wellcome, and the 10.13039/501100000770University of Manchester Strategic Fund. Funding for L.S. was received from 10.13039/501100012041Arthritis UK (ref. 21165). Funding for C.T.D and P.B. was received from the 10.13039/501100000265Medical Research Council (reference: MR/W019418/1). Funding was received from the Biotechnology and Biological Sciences Research Council (10.13039/501100000268BBSRC) Doctoral Training Program (BB/M011208/1) for a PhD studentship for A.-M.I. R.E.J. is supported through a Diabetes UK Harry Keen Intermediate Fellowship (reference: 20/0006263).

## Author contributions

Conceptualization, J.A.H. and S.M.R.; funding acquisition, J.A.H., R.D.U., and S.M.R.; methodology, Z.W., L.S., C.T.D., A.-M.I., T.Q., P.B., S.P., L.Z., L.J.B., R.E.J., N.A.H., and A.L.T.; data curation, Z.W., L.S., L.Z., and S.M.R.; formal analysis, Z.W., L.S., A.L.T., and R.D.U.; visualization, Z.W. and L.S.; manuscript preparation, Z.W.; manuscript review & editing, Z.W., L.S., A.L.T., R.D.U., J.A.H., and S.M.R. The manuscript was approved by all authors prior to submission.

## Declaration of interests

The authors declare no competing interests.

## STAR★Methods

### Key resources table

**Table:** 

REAGENT or RESOURCE	SOURCE	IDENTIFIER
**Antibodies**		

Mouse monoclonal anti-CD24-PE	BD Pharmingen	Cat# 555428 RRID: AB_395822
Rabbit polyclonal anti-EVC	Invitrogen	Cat# PA5-53412 RRID: AB_2641144
Rabbit polyclonal anti-Shh	Proteintech	Cat# 20697-1-AP RRID: AB_10694828
Rabbit polyclonal anti-PTCH1	Abcam	Cat# ab53715 RRID: AB_882208
Rabbit polyclonal anti-SMO	Proteintech	Cat# 20787-1-AP RRID: AB_2878740
Rabbit polyclonal anti-Gli1	Invitrogen	Cat# MA5-32553 RRID: AB_2809830
Goat polyclonal anti-Gli3	R & D Systems	Cat# AF3690 RRID: AB_2232499
Rabbit polyclonal anti-ARL13B	Proteintech	Cat# 17711-1-AP RRID: AB_2060867
Rat monoclonal anti-cytokeratin 19	DSHB	Cat# TROMA-III RRID: AB_2133570
Rabbit polyclonal anti-Collagen1	Proteintech	Cat# 14695-1-AP RRID: AB_2082037
Rabbit polyclonal anti-Collagen2	Proteintech	Cat# 28459-1-AP RRID: AB_2881147
Rabbit polyclonal anti-Collagen3	Proteintech	Cat# 22734-1-AP RRID: AB_2879158
Rabbit recombinant polyclonal anti-Sox9	Invitrogen	Cat# PA5-81966 RRID: AB_2789127
Rabbit polyclonal anti-SUFU	Proteintech	Cat# 26759-1-AP RRID: AB_2880625
Rabbit monoclonal anti-pSmad2	Cell Signaling Technology	Cat# 3108S RRID: AB_490941
Rabbit polyclonal anti-beta actin	Proteintech	Cat# 20536-1-AP RRID: AB_10700003
Mouse monoclonal anti-GAPDH	Sigma-Aldrich	Cat# CB1001 RRID: AB_2107426
Mouse monoclonal anti-acetylated alpha tubulin	Sigma-Aldrich	Cat# MABT868 RRID: AB_2819178
Mouse monoclonal Anti-γ-Tubulin	Sigma-Aldrich	Cat# T6557 RRID: AB_477584
Goat anti-Mouse IgG (H + L) Highly Cross-Adsorbed Secondary Antibody, Alexa Fluor™ 488	Invitrogen	Cat# A11029 RRID: AB_2534088
Goat anti-Rabbit IgG (H + L) Highly Cross-Adsorbed Secondary Antibody, Alexa Fluor™ 594	Invitrogen	Cat# A11037 RRID: AB_2534095
Goat Anti-Rat IgG H&L (Alexa Fluor® 594)	Abcam	Cat# Ab150160 RRID: AB_2756445
Donkey anti-Mouse IgG (H + L) Highly Cross-Adsorbed Secondary Antibody, Alexa Fluor™ 488	Invitrogen	Cat# A-21202 RRID: AB_141607
Donkey anti-Goat IgG (H + L) Highly Cross-Adsorbed Secondary Antibody, Alexa Fluor™ Plus 594	Invitrogen	Cat# A32758 RRID: AB_2762828
Goat Anti-Rabbit IgG Antibody, (H + L) HRP conjugate	Sigma-Aldrich	Cat# AP307P RRID: AB_92641
Goat Anti-Mouse IgG Antibody, (H + L) HRP conjugate	Sigma-Aldrich	Cat# AP308P RRID: AB_92635
Donkey anti-Goat IgG (H + L) Secondary Antibody, HRP	Invitrogen	Cat# PA1-28664 RRID: AB_10990162

**Oligonucleotides**		

Sequences of all primers and sgRNAs, as well as details of TaqMan Assays	Table S2	N/A

**Experimental models: Cell lines**		

Human NP cell line NP-nR105	van den Akker et al.^66^	N/A
NP-nR105-CRISPR-EVC-KO	This paper	N/A

**Chemicals, peptides, and recombinant proteins**		

Cas9 Nucleases	IDT	Cat# 1081058
DAPI	Thermo Fisher	Cat# 62247
SAG	Calbiochem	Cat# 56661
TGF-β3	Invitrogen	Cat# RP8600
SuperSignal West Pico PLUS Chemiluminescent Substrate	Thermo Fisher	Cat# 34580
Triton X-100	Sigma-Aldrich	Cat# T8787
TRIzol	Sigma-Aldrich	Cat# T9424

**Critical commercial assays**		

SERVA Purple Protein Quantification Kit	SERVA Electrophoresis GmbH	Cat# 39235.01
ABI high-capacity kit	Applied Biosystems	Cat# 4374967
TaqMan Fast Advanced Master Mix	Applied Biosystems	Cat# A44360
Fast SYBR Green Master Mix	Applied Biosystems	Cat# 4385610
RIPA buffer with Halt protease and phosphatase inhibitor cocktail	Thermo Fisher	Cat# 78440

**Deposited data**		

RNA-seq analysis	This paper	ArrayExpress: https://www.ebi.ac.uk/arrayexpress/; accession number E-MTAB-15392

**Software and algorithms**		

Adobe Illustrator	N/A	https://www.adobe.com
Adobe Photoshop	N/A	https://www.adobe.com
Crispor	N/A	http://crispor.tefor.net/
GraphPad Prism	N/A	https://www.graphpad.com
Huygens Pro (SVI)	N/A	https://svi.nl/
Illumina Bclfastq	N/A	https://www.illumina.com
Image Lab	N/A	https://www.bio-rad.com
ImageJ	N/A	https://imagej.net/ij/
ProteinPilot software (SCIEX)	N/A	https://sciex.com
RStudio	N/A	https://www.r-project.org
Snap gene	N/A	https://www.snapgene.com

### Experimental model and study participant details

#### Human and mouse IVD samples and tissue/cell preparation

Human embryonic and fetal spines were obtained with approval from the NHS Research Ethics Committee (Ref. No: 23/NW/0039; Early Pregnancy Tissue Collection) and with full informed written donor consent following medical or surgical pregnancy termination. Paediatric IVD samples were obtained from patients undergoing disc surgery for idiopathic scoliosis at Birmingham Royal Children’s Hospital with NHS Research Ethics Committee approval (Ref. No: 12/NW/0437) and full informed written donor consent. Adult degenerate cervical and lumbar IVD surgical samples were obtained from Salford Royal Hospital after informed written consent from patients undergoing disc replacement surgery for treatment of IVD degeneration and with the appropriate NHS Research Ethics Committee approvals in place (Ref: 17/LO/1408).

WT and global *Evc-/-* mice at postnatal day 0 (P0) (*n* = 3 per genotype) were generated as previously described.^31^ No sex differences have been observed in vertebrae morphology in early postnatal mice. Therefore, both sexes were used for our experiments. Breeding of genetically modified mice was conducted following guidelines set forth by King’s College London and the UK Home Office, with mice culled using Schedule one approved methods. Mice were maintained on a C57BL/6-CD1 mixed background to enhance viability of homozygous mutants, with mutants compared to littermate controls. Pups were fixed in 4% paraformaldehyde before dehydration through an ethanol series.

Human and mouse spine samples were carefully isolated under a stereomicroscope. Tissues were either processed as previously described to formalin-fixed, paraffin-embedded (FFPE) blocks from which 5 μm sections were prepared,^26^ or used for cell isolation. Human NP cells were isolated as previously described,^67^ and AF cells were also obtained from matched tissues where available. Histological grading of degeneration was performed on human spine samples based on standard haematoxylin and eosin (H&E) stained sections. Two independent observers assessed features of degeneration in the NP and AF according to a previously published scoring system.^68^ Briefly, scores were assigned out of 12, with 0–1 indicating no or minimal degeneration, 2–3 mild, 4–6 moderate, 7-8 advanced, and 9–12 severe degeneration.

Based on tissue availability, three independent human fetal spine samples at 14WPC were allocated to mass spectrometry analysis. Based on the study purpose and sample availability, three human fetal spine sections from different WPCs and one human paediatric spine section were allocated to immunofluorescence staining. For human adult samples (*n* = 58 patients), 58 NP samples and 14 matched AF samples were used for RT–qPCR analysis, and four samples with available NP tissue sections (*n* = 2, grade 4, moderate degeneration; *n* = 2, grade 11, severe degeneration) were allocated to immunofluorescence staining. Detailed information for all human disc samples used in this study is summarized in Table S1.

#### Cell culture

A previously described human NP cell line (NP-nR105)^66^ was cultured in DMEM high glucose (Sigma-Aldrich A5796) with 10% fetal bovine serum (Gibco A5256701), 100 units/mL antibiotic antimycotic solution (Sigma-Aldrich A5955), 10 μM ascorbate-2-phosphate (Sigma-Aldrich A8960), and 100 nM sodium pyruvate (Sigma-Aldrich S8636) at 37°C under 5% CO_2_. To induce ciliation and activate the Hh pathway, cells at approximately 80% confluence were switched to 0.5% FBS medium supplemented with small molecules. SAG (Calbiochem 56661) was used at 1 μM, and TGF-β3 (Invitrogen RP8600) at 10 ng/mL. Small-molecule exposures lasted 40 h, except for ICC analysis of ciliary Smo and Gli3, where exposure was 24 h.

### Method details

#### Mass spectrometry and bioinformatic analysis

Human fetal IVDs were microdissected and enzymatically digested using 0.1% Collagenase Type II and 0.02% Hyaluronidase in a non-enzymatic cell dissociation solution to release individual cells. Following digestion, cells were filtered and stained with anti-CD24-PE and DAPI (for viability) for 30 min at 4°C in the dark, then sorted by FACS to isolate CD24^+^ NCs and CD24^−^ SCs. Proteins were extracted from sorted cells using 1 M triethylammonium bicarbonate (TEAB) buffer supplemented with 0.1% (w/v) SDS and quantified using the SERVA Purple Protein Quantification Kit. Samples (10 μg protein) were reduced, alkylated, and digested with trypsin prior to labeling with isobaric tags for relative and absolute quantitation (iTRAQ).^69^ Labelled samples were pooled and fractionated using high pH reverse-phase liquid chromatography (LC) followed by nano-flow LC, and tandem mass spectrometry (MS/MS) using a SCIEX 6600 TripleTOF mass spectrometer as previously described.^70^ Protein identification and peptide quantification were performed using ProteinPilot software (SCIEX). Quantitative analysis was subsequently carried out using SeaMASS software based on the ProteinPilot peptide summary as described^71^ (Figure 1A).

#### Immunofluorescence

Tissue sections were dewaxed and antigen retrieval was performed with Tris-EDTA buffer (pH 9.0). Permeabilization was conducted using 0.25% Triton-X in PBS (PBST), followed by blocking with 4% BSA in 0.1% PBST. Slides were incubated with primary antibodies (see key resources table) in blocking buffer at 4°C overnight. After three PBS washes, secondary antibodies were applied in blocking buffer for 1 h at room temperature in the dark, followed by DAPI counterstaining and a 10-min soak in 50 mM ammonium chloride. Finally, slides were mounted with VECTASHIELD. Images were acquired on an Olympus IX83 inverted microscope with Cyan Lumencor LED excitation and a Retiga R6 (Qimaging) CCD camera, using 0.2 μm Z optical spacing. Raw images were deconvolved with Huygens Pro (SVI), followed by maximum intensity projection. Representative images were uniformly processed in ImageJ, with processed projections presented in final results and used for statistical analysis.

#### RNA extraction and RT-qPCR

RNA samples were prepared using TRIzol reagent, and cDNA synthesis was performed using the ABI high-capacity kit with RNase inhibitor. PCR reactions were conducted in triplicate using either TaqMan Fast Advanced Master Mix or Fast SYBR Green Master Mix on an ABI StepOnePlus real-time PCR system. Sequences of all primers and details of TaqMan assays are provided in Table S2. RT-qPCR data were analyzsed using the 2^−ΔCT^ or 2^−ΔΔCT^ methods, with normalization to the average expression of the reference genes *GAPDH* or *MRPL19*, except for the analysis of *GLI3* gene expression levels in WT and *EVC-/-* cells treated with or without SAG, which was normalized to *GAPDH* alone.

#### Histological staining

Mouse spine sections were dewaxed and stained sequentially with 1% alcian blue (pH 2.5) for 20 min, washed in running tap water for 5 min, 0.04% fast green for 20 min, washed again in running tap water for 5 min, and 0.1% picrosirius red for 30 min, followed by two rinses in 1% acidified water for a total of 5 min. Images were captured using a 3D-Histech Pannoramic-250 microscope slide-scanner at the Bioimaging Facility, University of Manchester. Spine sections from three WT and *Evc*-/- mice were stained, with one representative image shown in the results.

#### CRISPR knockout

Human NP *EVC* knockout cells were generated using CRISPR/Cas9 targeting *EVC* exon 4 with an sgRNA (TGTCATCGCTGGTGGCCGAG TGG) designed in-house and synthesized by Integrated DNA Technologies (IDT). The sgRNA was delivered via electroporation (1400 V, 20 ms, 2 pulses). Monoclonal lines were isolated by serial dilution and expanded. Knockout validation was confirmed using Sanger sequencing, IF, and Western blot analysis. Sequences of genotyping primers are provided in Table S2.

#### Immunocytochemistry

ICC was performed on cells plated in 8-well chambered coverslips (Ibidi). Cells were fixed in 4% PFA at room temperature for 10 min, followed by post-fixation in −20°C methanol for 5 min. After permeabilization with 0.1% PBST, cells were blocked in 2% BSA in 0.1% PBST. Primary antibodies diluted in blocking buffer were applied overnight at 4°C, followed by incubation with secondary antibodies in blocking buffer at room temperature for 1 h in the dark. Finally, cells were counterstained with DAPI and mounted using ibidi Mounting Medium.

#### Western blot

Protein samples were prepared in RIPA buffer with Halt protease and phosphatase inhibitor cocktail (Thermo Fisher 78440, 1:100). Protein concentration was determined using the Pierce BCA Protein Assay Kit (Thermo Fisher 23227) according to the manufacturer’s instructions. For SDS-PAGE, 10 μg of protein was loaded onto precast gels (Mini Gel Tank system, Thermo Fisher A25977), followed by wet transfer onto a 0.45 μm PVDF membrane (Invitrolon LC2005) using the Mini Blot Module (Invitrogen B1000). Membranes were blocked with 5% non-fat milk in TBS with 0.1% Tween 20 (TBST) and incubated with primary antibodies diluted in either blocking buffer or TBST with 5% BSA for 1 h at room temperature or overnight at 4°C. After washing, membranes were incubated with HRP-conjugated secondary antibodies in blocking buffer for 1 h at room temperature. Signal was developed using SuperSignal West Pico PLUS Chemiluminescent Substrate (Thermo Fisher 34580) and visualised on a Bio-Rad Chemidoc MP Imager. Western blot band intensity was quantified using ImageJ.

#### Bulk RNA sequencing and bioinformatic analysis

RNA samples from WT and *EVC*-/- human NP cells with or without SAG treatment (*n* = 4) were prepared using TRIzol. The Genomic Technologies Core Facility, University of Manchester, performed the Illumina Library prep and sequencing using Illumina NovaSeq6000 system. The sequenced data were processed using the Illumina Bclfastq 2.20.0.422 software, and quality control was performed using FastqScreen, FastqStrand, FastQC, MultiQC, QualiMap, and RSeQC geneBody_coverage. Raw gene counts were normalized, and differential expression analysis was conducted using DESeq2 with the Benjamini-Hochberg false discovery rate (FDR) multiple testing correction. GO analysis was performed using Enrichr.^72^ A threshold cut-off of log_2_ fold change (LogFC) > ± 0.5 and p-adjust value <0.05 was generally applied. Data were visualised using ComplexHeatmap,^73^ Hiplot,^74^ ggplot2^75^ and EnhancedVolcano^76^ plots.

### Quantification and statistical analysis

Lengths of vertebral bodies and IVDs in mouse spine sections were measured using the line tool in CaseViewer. Fluorescence intensity was quantified within regions of interest (ROIs) using ImageJ. For the analysis of ciliation proportions and ciliary length, two ciliary markers, Acet-Tub and Arl13b, were used to label primary cilia. Cells displaying positive signals for both markers were recorded as ciliated, and the ciliary length was manually measured using the segmented line tool in ImageJ. NP cells having multiple primary cilia were counted as a single ciliated cell, but all ciliary lengths were recorded for subsequent length analysis. For quantification of ciliary Smo fluorescence intensity, a mask was created by thresholding or manually delineating around each primary cilium labelled with the ciliary axoneme marker Acet-Tub in the FITC channel. The mean intensity of Smo was then measured in the TRITC channel. For ciliary Gli3 analysis, a circle mask of identical size (10 × 10) was manually created at each ciliary tip. The placement of the mask was guided by the basal body marker γ-Tub and the ciliary axoneme marker Acet-Tub (to distinguish the ciliary base and the ciliary tip) in the FITC channel, and the mean intensities of Gli3 at each ciliary tip were measured in the TRITC channel. Statistical analyses were conducted in GraphPad Prism or R, based on a minimum of three independent biological replicates. Pearson correlation, unpaired t-tests and paired t-tests, one-way ANOVA, and two-way ANOVA were used as appropriate. Data are presented as mean ± SEM, with *p* < 0.05 deemed statistically significant.

## Supplemental information

Document S1. Figures S1–S7 and Tables S1 and S2

Data S1. Proteomics analysis
