# Supplementary material for: EVC protein regulates Sonic hedgehog signaling during human intervertebral disc development and degeneration
Source: iScience. 2025 Dec 4;29(1):114290. doi: 10.1016/j.isci.2025.114290 (PMC12808898; doi:10.1016/j.isci.2025.114290)
Supplement: Document S1. Figures S1–S7 and Tables S1 and S2 [file mmc1.pdf]

iScience, Volume *29*

Supplemental information

EVC protein regulates Sonic hedgehog signaling during human intervertebral disc development and degeneration

Zihan Wu, Lizzy Shaw, Christabel T. Dube, Andra-Maria Ionescu, Tengyang Qiu, Anna L. Tierney, Pauline Baird, Sonal Patel, Leo A. H. Zeef, Lindsay J. Birchall, Rachel E. Jennings, Neil A. Hanley, Richard D. Unwin, Judith A. Hoyland, and Stephen M. Richardson

Supplementary Figures

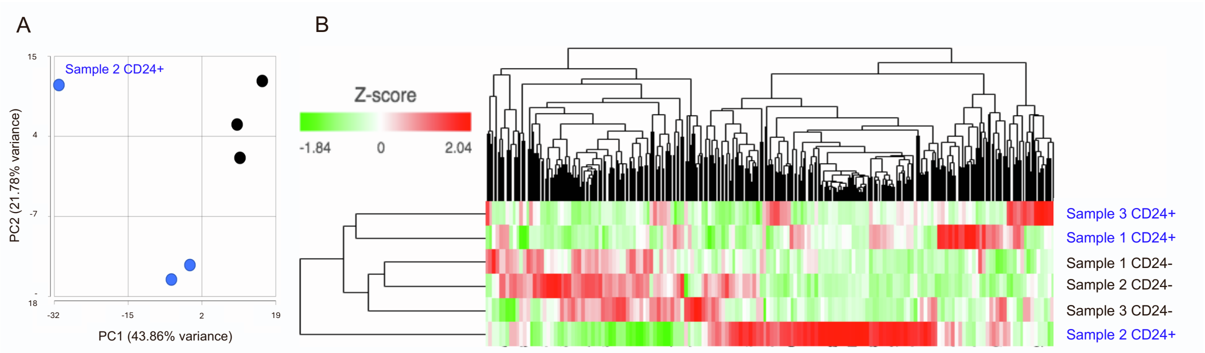

FIGURE S1. Proteomic analysis of human fetal NCs, related to Figure 1. (**A**) PCA plot illustrating protein expression distributions between CD24+ NC and CD24- SC populations. (**B**) Heatmap depicting protein profile among individual samples.

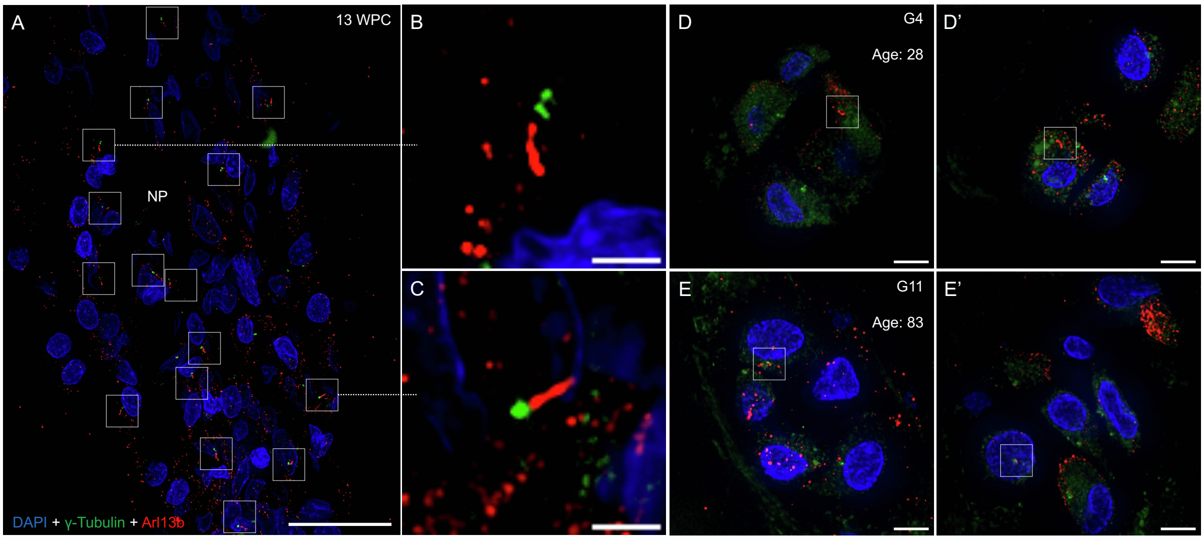

FIGURE S2. IF staining of primary cilia in human spine sections at different developmental and degenerative stages, related to Figure 2. (**A**) 13WPC NP with higher magnification in panels (**B**) and (**C**); (**D-D'**) Grade 4 degenerate NP; (**E-E'**) Grade 11 degenerate NP. Primary cilia are highlighted with white squares. Green = γ-Tubulin; Red = Arl13b; Blue = DAPI-stained nuclei. Scale bar = 50 μm in A, D, D', E and E'; 2 μm in B and C.

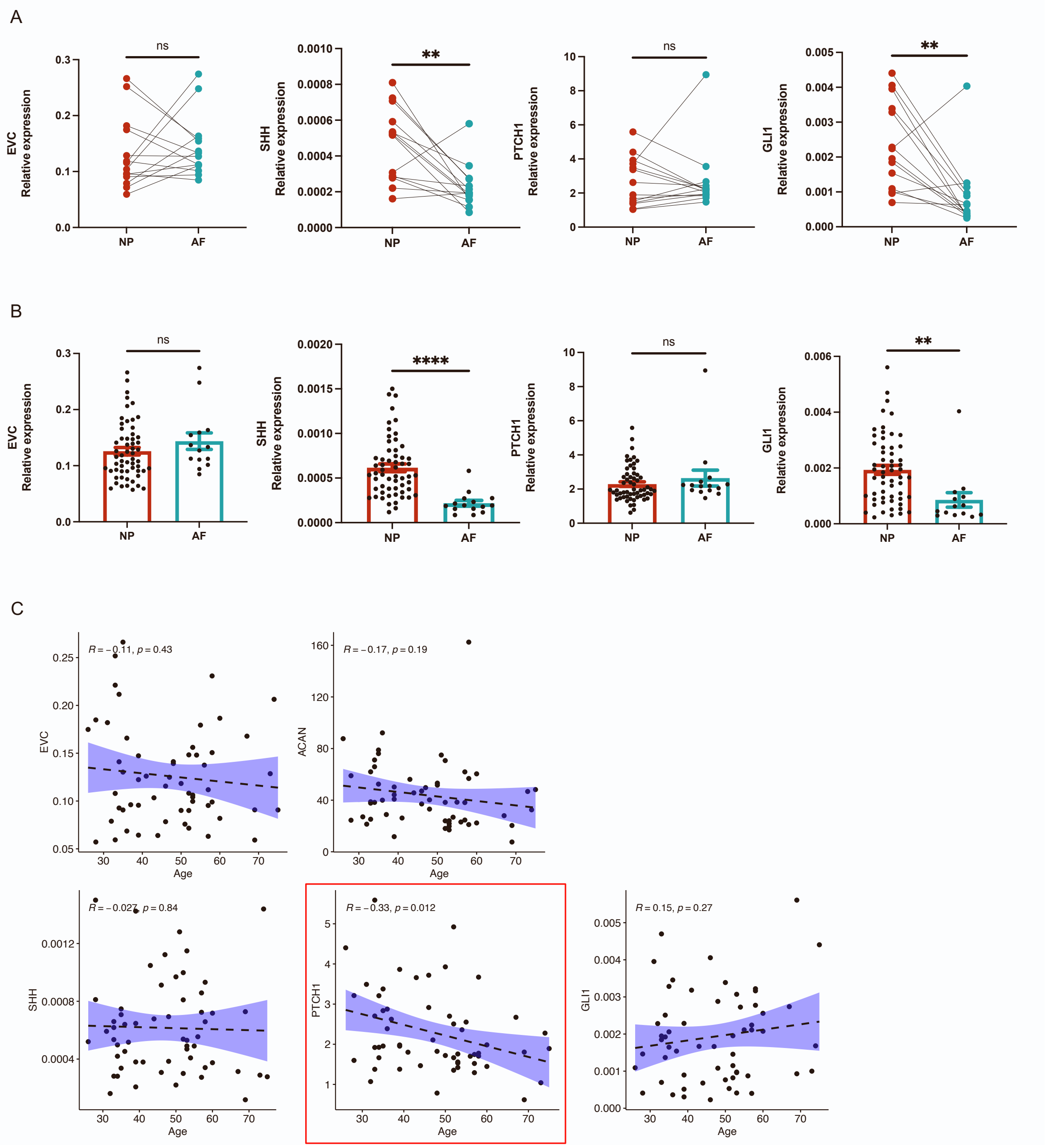

FIGURE S3. Gene expression analysis of EVC and Shh pathway components in human NP and AF samples, related to Figure 2. (**A**) Paired comparison of *EVC*, *SHH*, *PTCH1*, and *GLI1* gene expression between matched NP and AF samples (n=14), presented as before-after plots. (**B**) Comparison of gene expression levels between all NP (n=58) and AF (n=14) samples, displayed as mean ± SEM. (**C**) Correlation analysis between donor age and gene expression levels of *EVC*, *SHH*, *PTCH1*, *GLI1* and *ACAN* in degenerate NP samples (n=58). Relative gene expression was calculated using the $2^{-\Delta Ct}$ method and normalized to the average of *MRPL19* and *GAPDH*. Statistical significance was determined using unpaired t-test for group comparisons and simple linear regression for correlation analyses, with R values indicating correlation strength. Each dot represents an individual sample; dotted lines depict predicted trends with 95% confidence intervals. (**) p < 0.01; (****) p < 0.0001.

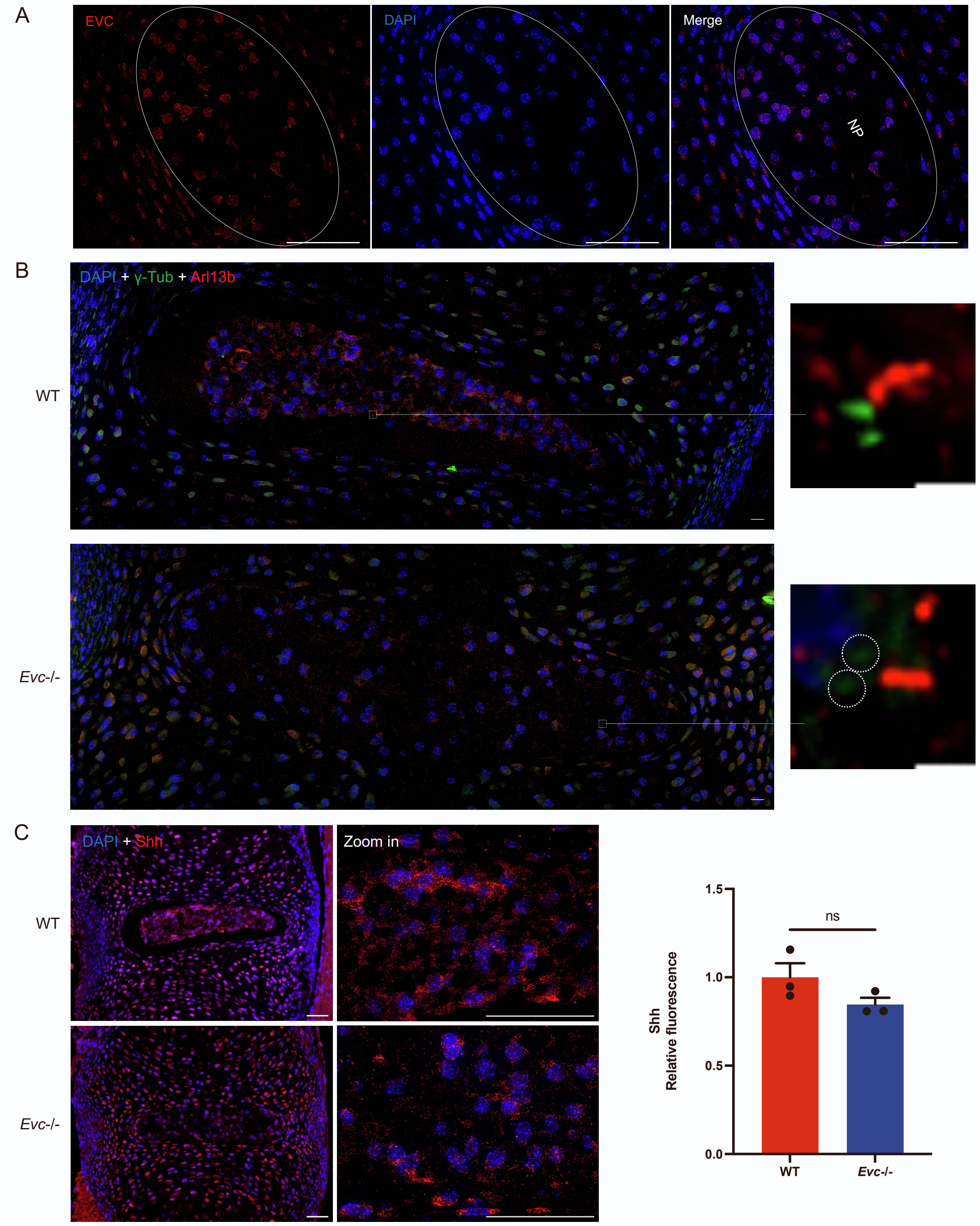

FIGURE S4. EVC expression pattern, primary cilium visualization, and Shh expression analysis in mouse IVDs, related to Figure 3. (**A**) IF staining of EVC in P0 WT mouse NP. (**B**) IF staining of primary cilia in WT and EVC-/- mouse IVD sections, with higher magnification images highlighting well-defined cilia. (**C**) IF staining and quantification of Shh expression in IVD sections from WT and EVC-/- mice (n=3). Fluorescence intensity normalised to WT averages and presented as mean ± SEM; statistical significance assessed using unpaired t-test. Scale bar = 50 μm for all images, except 1 μm in zoom-in images in (B).

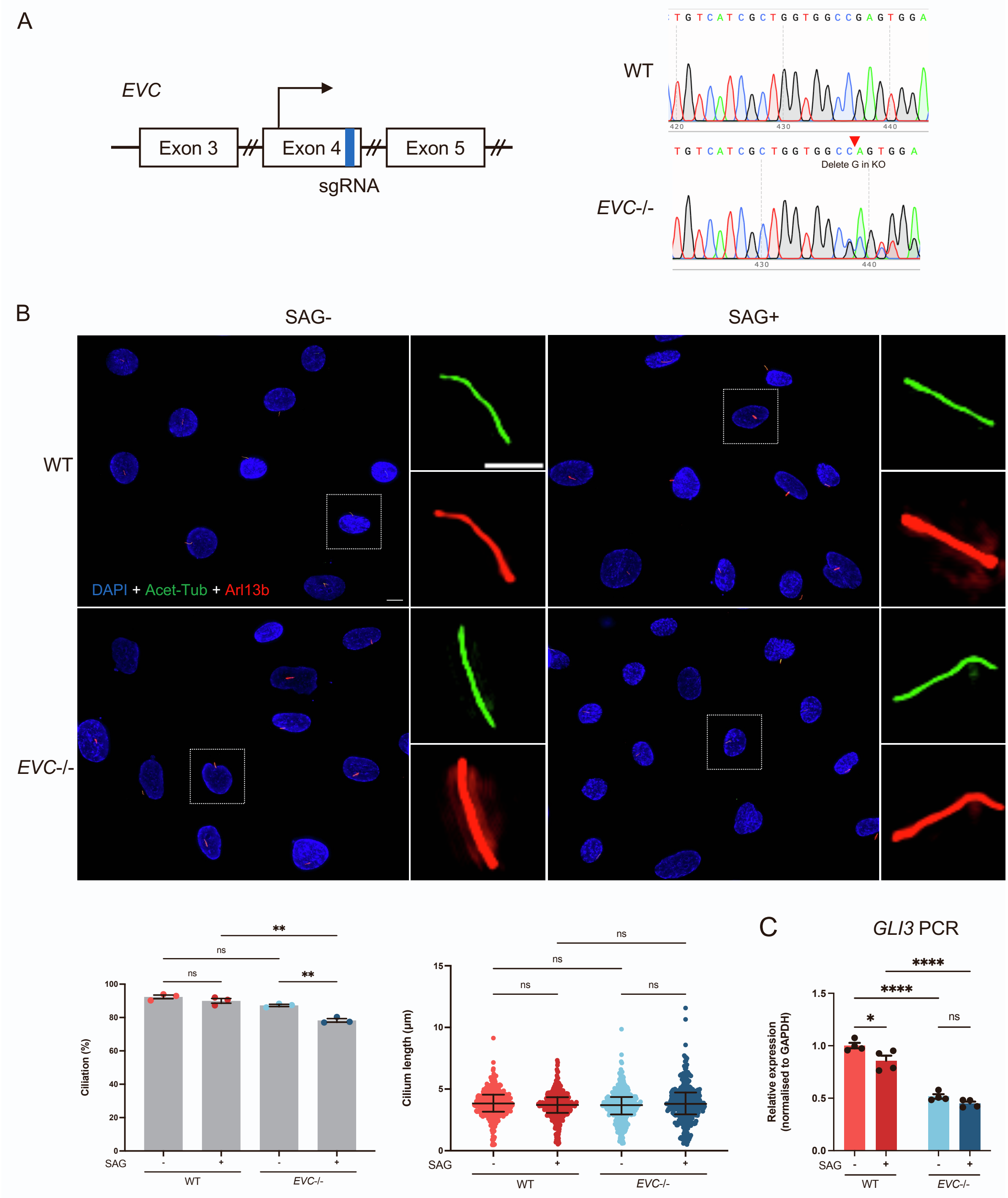

FIGURE S5. Generation of human EVC knockout NP cells and characterization of primary cilium phenotype, related to Figure 4. (**A**) Schematic representation of the human *EVC* exome highlighting the CRISPR/Cas9-targeted exon, accompanied by Sanger sequencing showing the edited site. (**B**) IF staining of primary cilium in human WT and EVC-/- cells with or without SAG activation, and quantitative analysis of cilium length (n=325, 357, 355 and 341 cilia in WT SAG-, WT SAG+, EVC SAG-, and EVC SAG+, respectively) and ciliation frequency (n=3 independent experiments). Scale bar = 10 µm in DAPI-stained panels and 2 µm in magnified images. (**C**) RT-qPCR analysis of *GLI3* expression in WT and EVC-/- cells (n=4), with or without SAG treatment. Gene expression levels were normalized to *GAPDH* and presented relative to WT SAG- averages. Data are presented as mean ± SEM. Statistical significance was determined using two-way ANOVA followed by Tukey's multiple comparison test. (*) $p < 0.05$; (**) $p < 0.01$; (****) $p < 0.0001$.

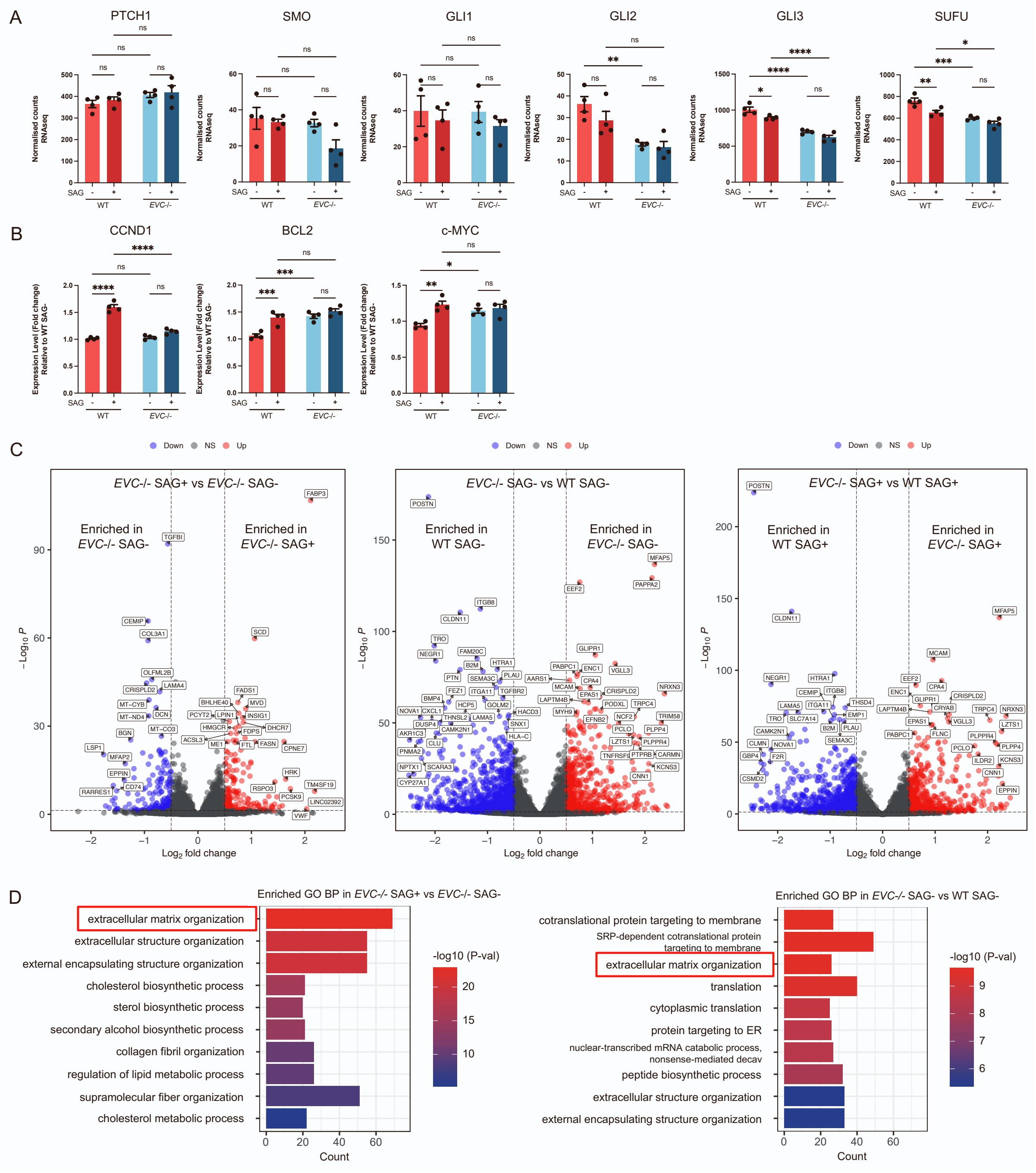

FIGURE S6. RNA-seq analysis of Shh pathway components, target genes, Enriched GO BP, and differentially expressed genes, related to Figure 5. (**A**) RNA-seq analysis of *PTCH1*, *SMO*, *GLI1*, *GLI2*, *GLI3* and *SUFU* expression in WT and EVC-/- cells with and without SAG treatment. Normalised count values are shown and presented as mean ± SEM (n=4). (**B**) RNA-seq analysis of *CCND1*, *BCL2*, and *c-MYC* expression in WT and EVC-/- cells with and without SAG treatment. Data are presented as mean ± SEM, normalized to WT SAG- sample 1 (n=4). (**C**) Volcano plot illustrating the differentially expressed genes in all paired groups. (**D**) Top 10 terms of Enriched GO BP analysis of differentially expressed genes in all paired groups. LogFC cut off = 0.5, p-value cut off = 0.05. Terms are ranked by p-adjust value. Statistical significance was determined by two-way ANOVA followed by Tukey's multiple comparison test. (*) $p < 0.05$; (**) $p < 0.01$; (***) $p < 0.001$; (****) $p < 0.0001$.

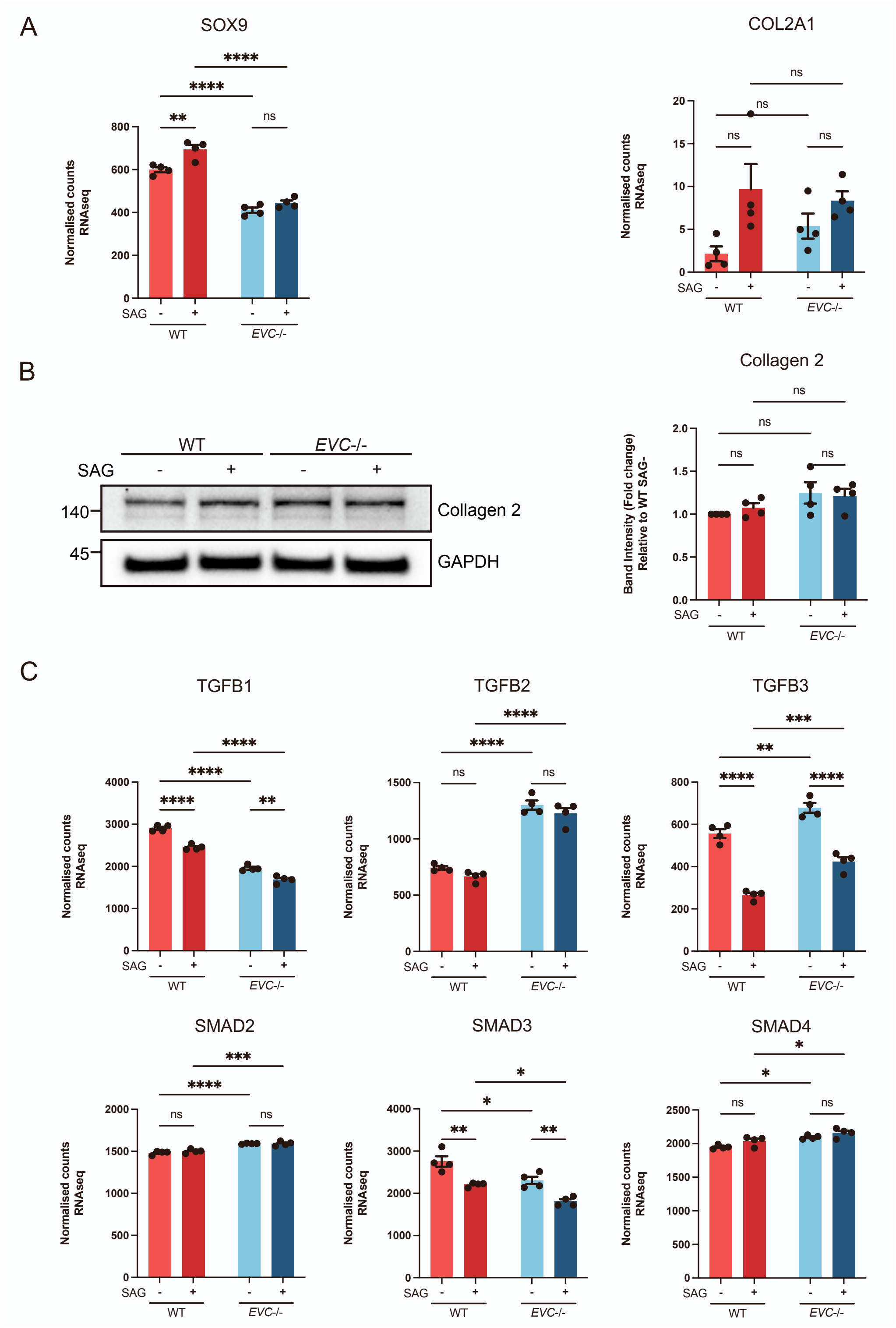

FIGURE S7. Analysis of SOX9, COL2A1, Collagen 2, and TGF-β signaling components, related to Figure 6. (**A**) RNA-seq analysis of *SOX9* and *COL2A1* expression in WT and EVC-/- cells with or without SAG treatment (n=4). (**B**) Western blot analysis of Collagen 2 protein levels in WT and EVC-/- cells with or without SAG treatment (n=4). (**C**) RNA-seq analysis of TGF-β signaling components, including *TGFB1*, *TGFB2*, *TGFB3*, *SMAD2*, *SMAD3* and *SMAD4*, in WT and EVC-/- cells with or without SAG activation (n=4). Normalised count values are shown for RNA-seq analyses. Protein expression levels were normalized to WT SAG- samples. Data are presented as mean ± SEM. Statistical significance was determined by two-way ANOVA followed by Tukey's multiple comparison test. (*) $p < 0.05$; (**) $p < 0.01$; (***) $p < 0.001$; (****) $p < 0.0001$.

Supplementary Tables

Donor age	Donor sex	Histological grade of degeneration	Matched AF	LC-MS/MS	IF	RT-qPCR
**Fetal**						
7 WPC	Unknown	0			X	
13 WPC	Unknown	0			X	
14 WPC	Unknown	0			X	
14 WPC	Unknown	0		X		
14 WPC	Unknown	0		X		
14 WPC	Unknown	0		X		
**Paediatric**						
10 year	Female	0			X	
**Adult**						
26 years	Female	4	X		X	X
28 years	Female	5				X
28 years	Male	11				X
28 years	Female	4			X	
31 years	Male	9	X			X
32 years	Male	7	X			X
33 years	Male	5				X
33 years	Male	11	X			X
33 years	Male	6	X			X
33 years	Male	10				X
34 years	Female	8				X
34 years	Male	9				X
34 years	Female	7				X
35 years	Female	4				X
35 years	Male	8	X			X
35 years	Female	6				X
36 years	Female	10				X
36 years	Female	7				X
37 years	Female	10	X			X
39 years	Female	6				X
39 years	Female	7				X
39 years	Male	6				X
39 years	Female	6				X
41 years	Male	6				X
43 years	Female	6				X
44 years	Female	11				X
46 years	Male	9	X			X
46 years	Female	11				X
47 years	Male	8				X
48 years	Male	9				X
48 years	Male	8				X
50 years	Male	8	X			X
50 years	Female	11				X
51 years	Female	7				X
52 years	Female	8				X
52 years	Female	3	X			X
52 years	Female	8				X
52 years	Female	5				X
53 years	Male	10				X
53 years	Male	7				X

53 years	Male	4	X			X
53 years	Female	7				X
54 years	Female	7				X
55 years	Female	11				X
56 years	Female	6				X
57 years	Male	8				X
57 years	Male	11				X
57 years	Female	7	X			X
58 years	Male	7				X
58 years	Female	10				X
58 years	Female	10				X
60 years	Female	4				X
60 years	Male	6				X
67 years	Male	9				X
69 years	Male	4				X
69 years	Male	7				X
72 years	Male	11			X	
73 years	Female	8	X			X
74 years	Male	5				X
75 years	Female	4	X			X
83 years	Female	11			X	

Primer sequence / TaqMan assay / sgRNA	SOURCE	IDENTIFIER
TaqMan Gene expression Assay for EVC	TaqMan	Cat# Hs00205772_m1
TaqMan Gene expression Assay for GLI1	TaqMan	Cat# Hs00171790_m1
TaqMan Gene expression Assay for GLI3	TaqMan	Cat# Hs00609233_m1
SHH PCR Fwd: 5'-ACGAGTCCAAGGCACATATCCA-3'	IDT	N/A
SHH PCR Rev: 5'-GCTCAGGTCCTTCACCAGCTT-3'	IDT	N/A
PTCH1 PCR Fwd: 5'-GTCGCACAGAACTCCACTCA-3'	IDT	N/A
PTCH1 PCR Rev: 5'-GTCAGAGAAGGATTTCAGGATGTC-3'	IDT	N/A
GAPDH PCR Fwd: 5'-CTCCTCTGACTTCAACAG-3'	IDT	N/A
GAPDH PCR Rev: 5'-CGTTGTCATACCAGGAAA-3'	IDT	N/A
MRPL19 PCR Fwd: 5'-CCACATTCCAGAGTTCTA-3'	IDT	N/A
MRPL19 PCR Rev: 5'-CCGAGGATTATAAAGTTCAAA-3'	IDT	N/A
ACAN PCR Fwd: 5'-GGCTTCCACCAGTGTGAC-3'	IDT	N/A
ACAN PCR Rev: 5'-GTGTCTCGGATGCCATACG-3'	IDT	N/A
EVC sgRNA: 5'-TGTCATCGCTGGTGGCCGAG TGG-3'	IDT	N/A
EVC genotyping PCR Fwd: 5'-GGACGGAAACTCTGTGGTGT-3'	IDT	N/A
EVC genotyping PCR Rev: 5'-TGCTGAACTTGTCCCAGGAT-3'	IDT	N/A
