# Supplementary material for: EVC protein regulates Sonic hedgehog signaling during human intervertebral disc development and degeneration
Source: iScience. 2025 Dec 4;29(1):114290. doi: 10.1016/j.isci.2025.114290 (PMC12808898; doi:10.1016/j.isci.2025.114290)
Supplement: Data S1. Proteomics analysis [file mmc2.pdf]

Figure 4B

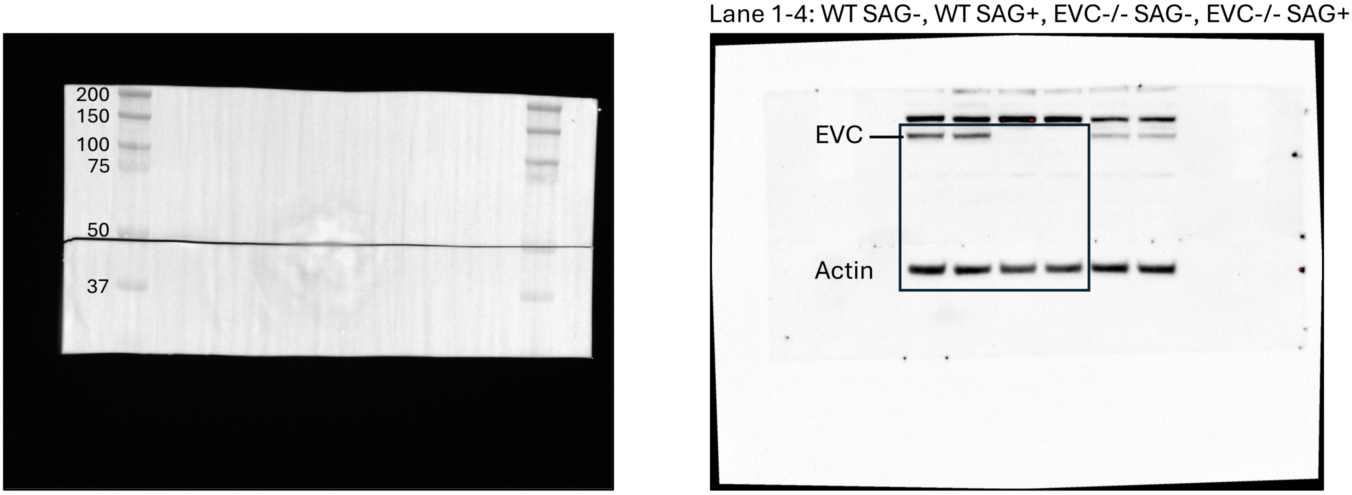

Figure 4C

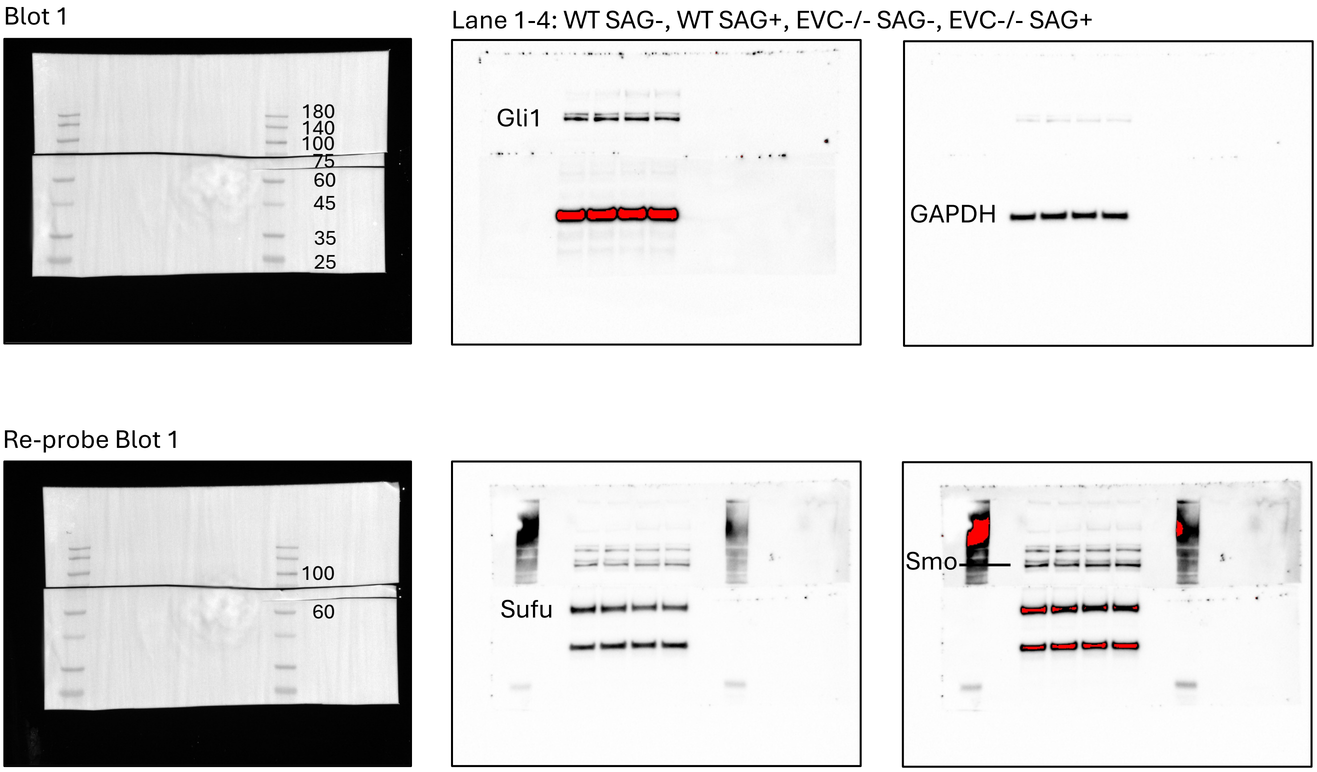

Figure 4B continued

Blot 2

Lane 1-4: WT SAG-, WT SAG+, EVC-/- SAG-, EVC-/- SAG+

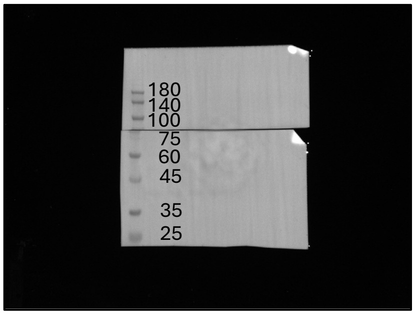

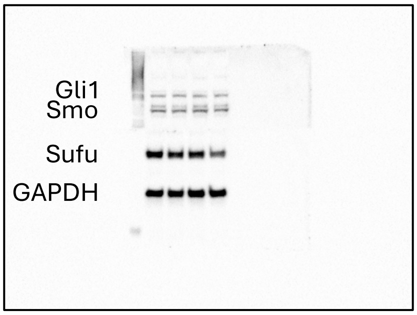

Blot 3

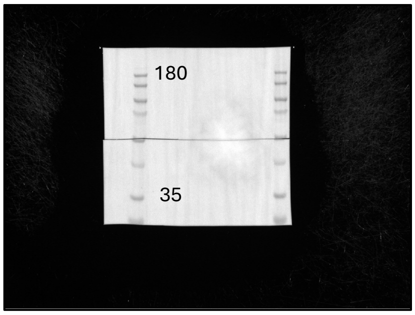

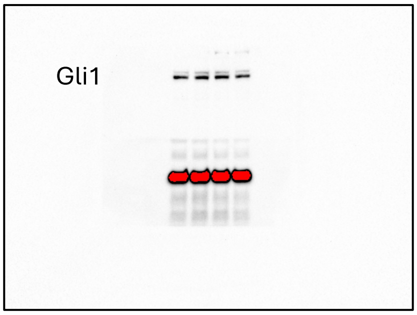

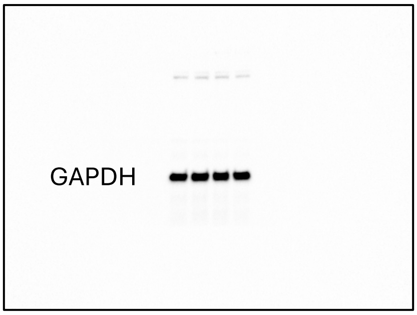

Re-probe blot 3

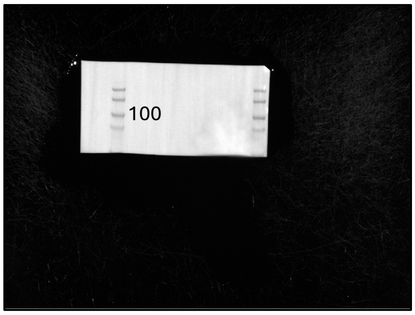

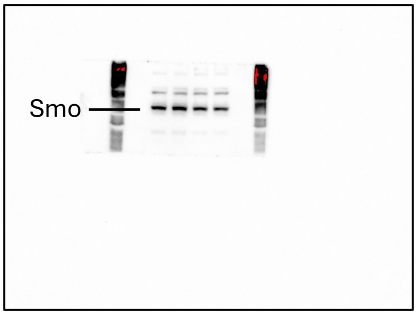

Blot 4 (Sufu_n2)

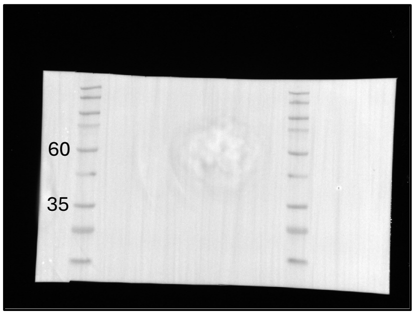

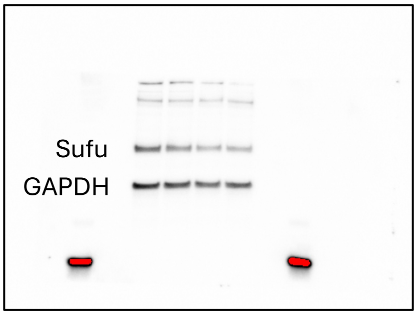

Figure 4E

Blot 1

Lane 1-4: WT SAG-, WT SAG+, EVC-/- SAG-, EVC-/- SAG+

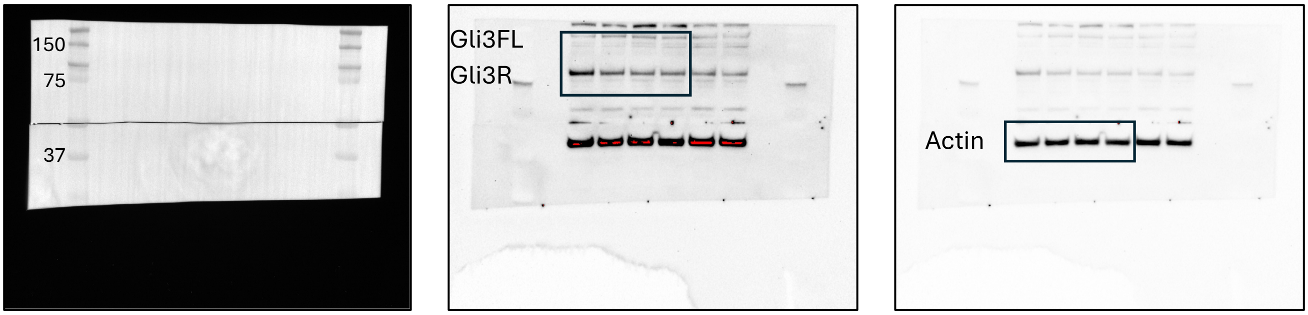

Blot 2

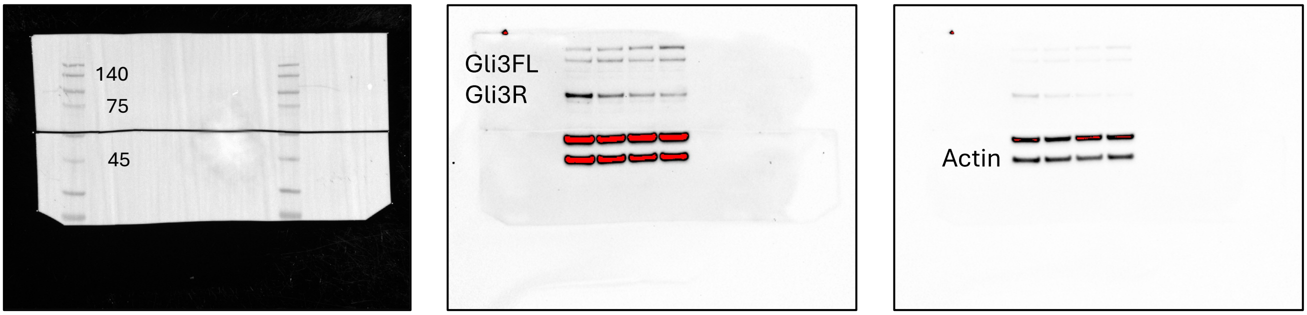

Blot 3

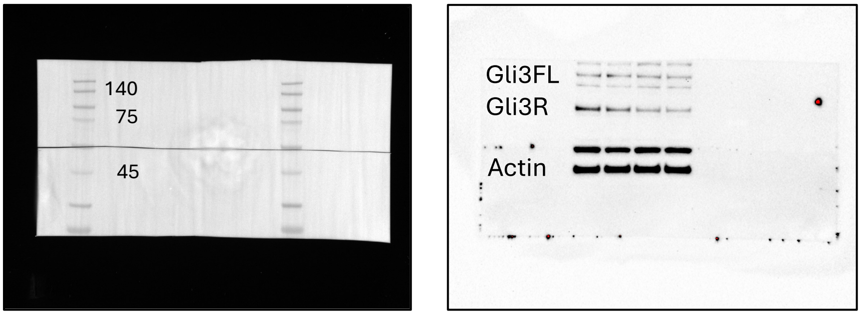

Figure 6A

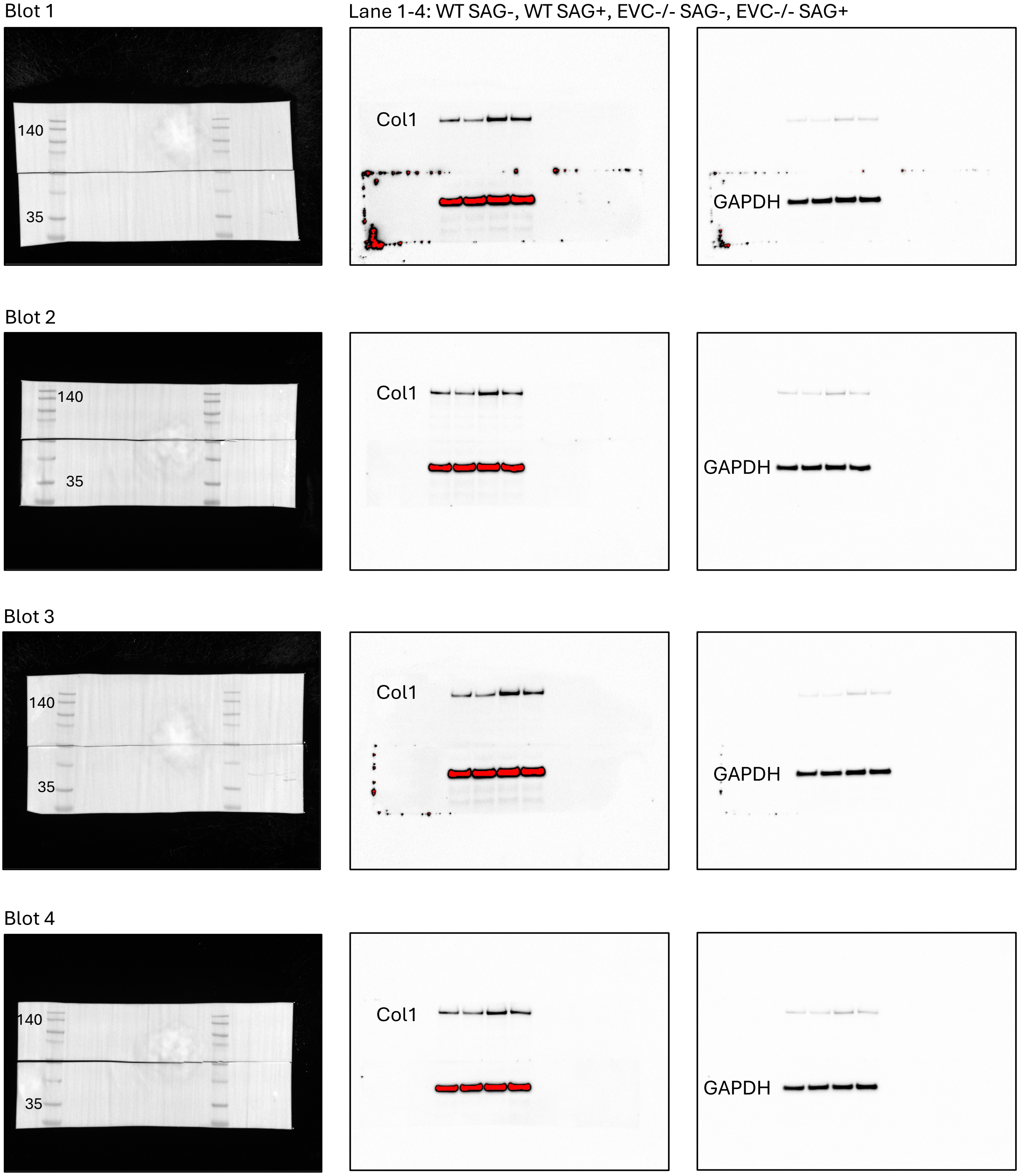
Blot 1
Lane 1-4: WT SAG-, WT SAG+, EVC-/- SAG-, EVC-/- SAG+
140
35
Col1
GAPDH
Blot 2
140
35
Col1
GAPDH
Blot 3
140
35
Col1
GAPDH
Blot 4
140
35
Col1
GAPDH

Figure 6B

Blot 1

Lane 1-4: WT SAG-, WT SAG+, EVC-/- SAG-, EVC-/- SAG+

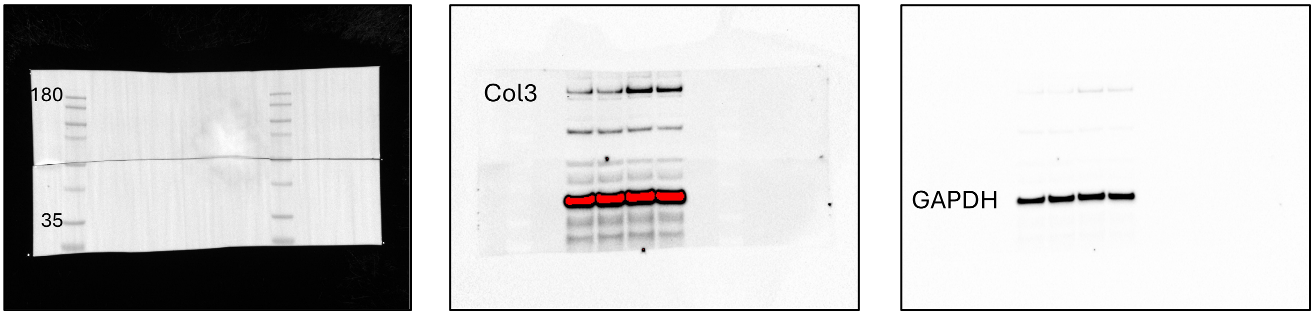

Blot 2

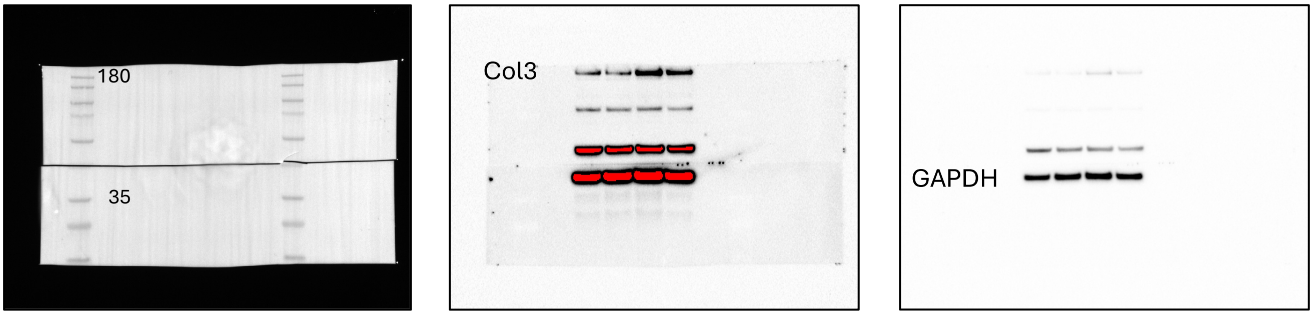

Blot 3

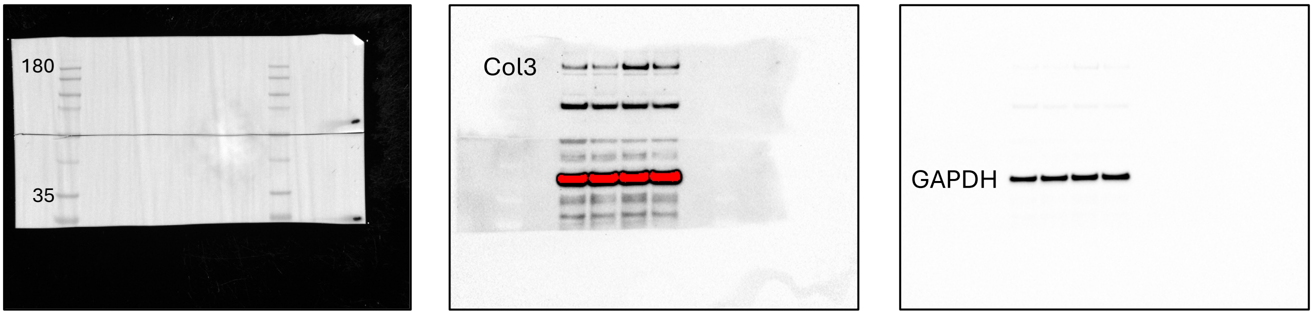

Figure 6C

Blot 1

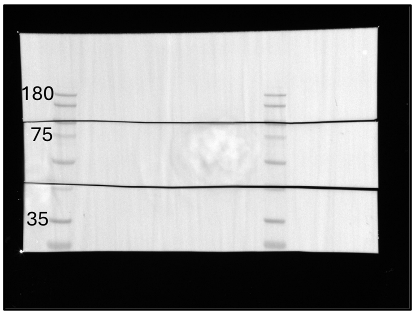

Lane 1-4: WT SAG-, WT SAG+, EVC /- SAG-, EVC-/- SAG+

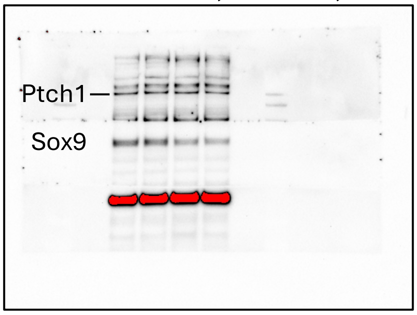

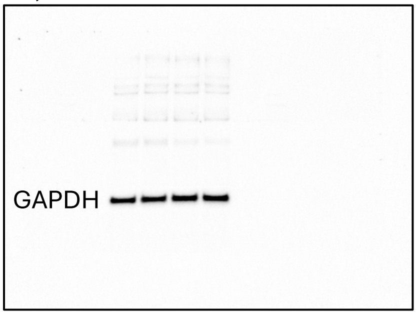

Blot 2 (n2 & n3)

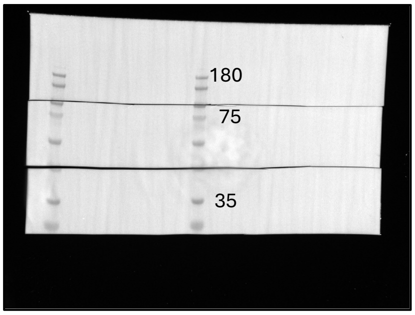

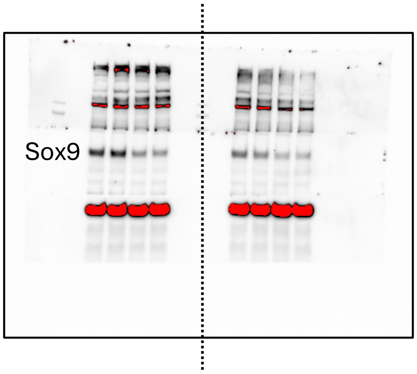

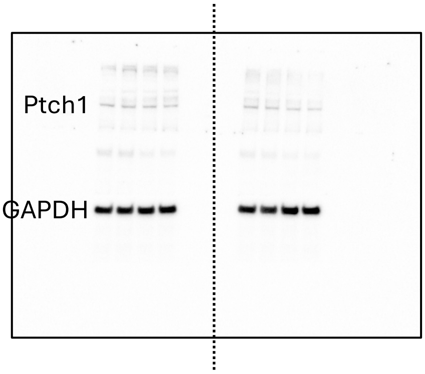

Figure 6D

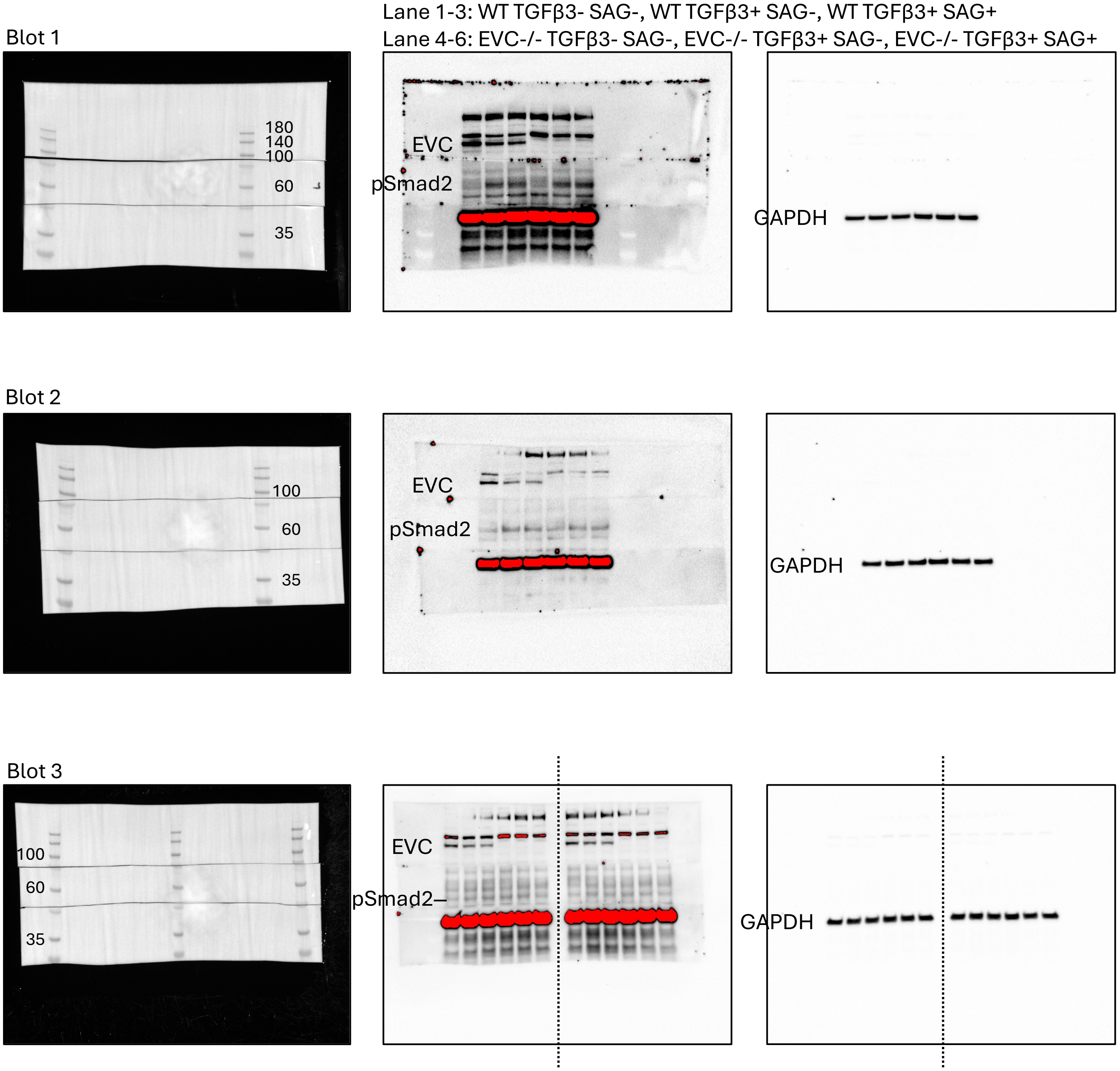
Lane 1-3: WT TGFβ3- SAG-, WT TGFβ3+ SAG-, WT TGFβ3+ SAG+
Lane 4-6: EVC-/- TGFβ3- SAG-, EVC-/- TGFβ3+ SAG-, EVC-/- TGFβ3+ SAG+
Blot 1
180
140
100
60
35
EVC
pSmad2
GAPDH
Blot 2
100
60
35
EVC
pSmad2
GAPDH
Blot 3
100
60
35
EVC
pSmad2
GAPDH

Figure 6E & 6F

Lane 1-3: WT TGFβ3- SAG-, WT TGFβ3+ SAG-, WT TGFβ3+ SAG+
Lane 4-6: EVC-/- TGFβ3- SAG-, EVC-/- TGFβ3+ SAG-, EVC-/- TGFβ3+ SAG+

Blot 1

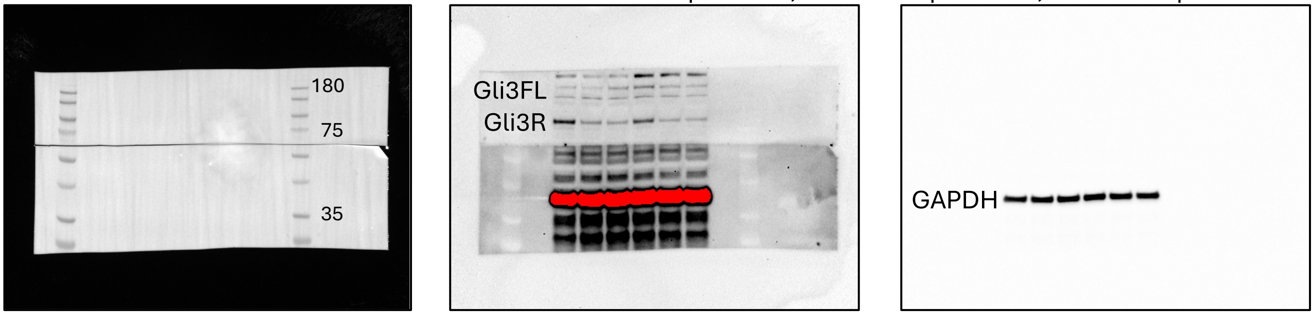

Blot 2

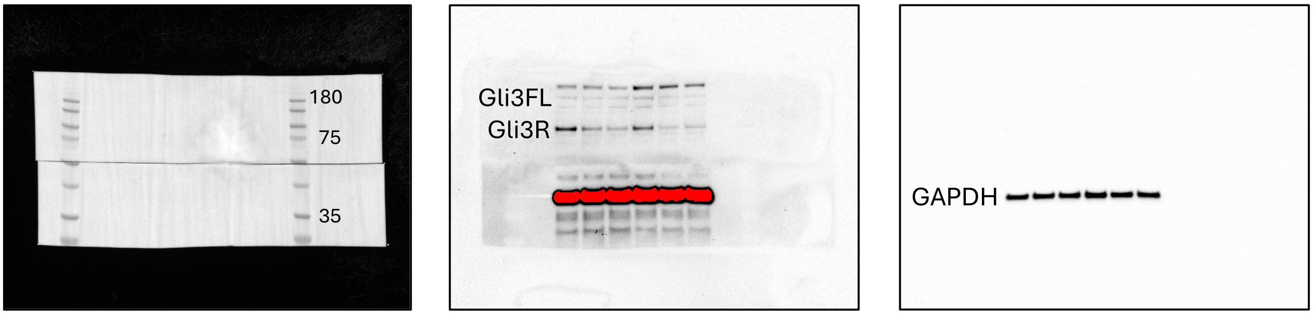

Re-probe Blot 2

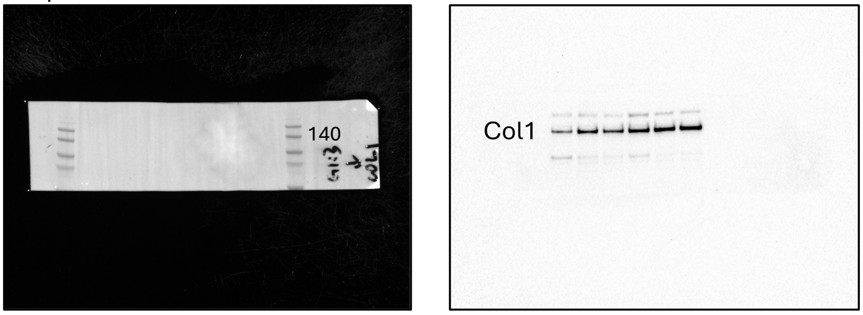

Figure 6E & 6F

Blot 3

Lane 1-3: WT TGFβ3- SAG-, WT TGFβ3+ SAG-, WT TGFβ3+ SAG+
Lane 4-6: EVC-/- TGFβ3- SAG-, EVC-/- TGFβ3+ SAG-, EVC-/- TGFβ3+ SAG+

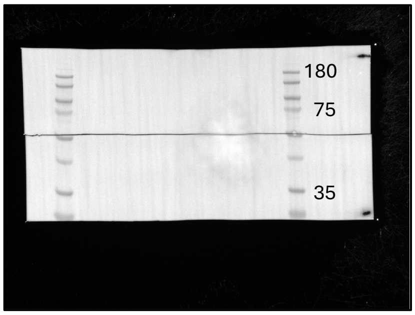

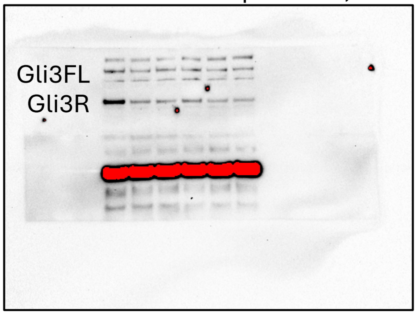

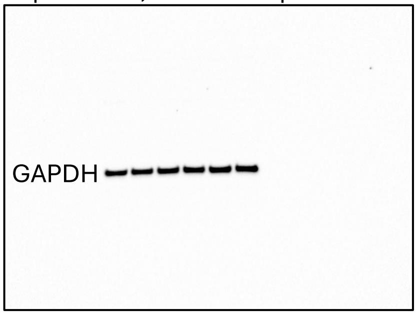

Re-probe Blot 3

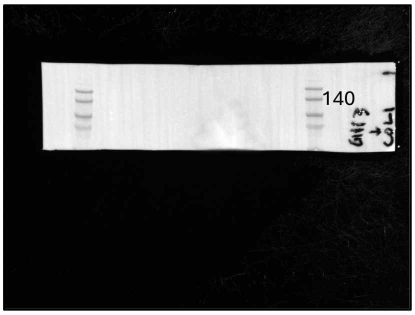

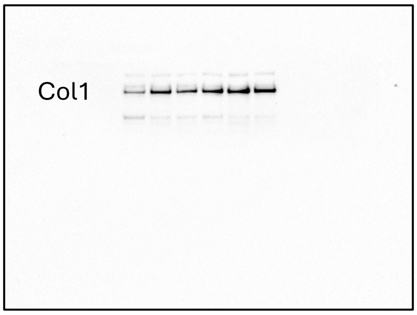

Blot 4 (Col1_n1)

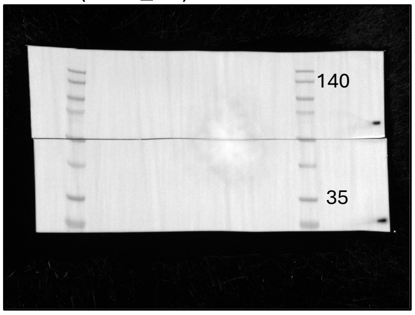

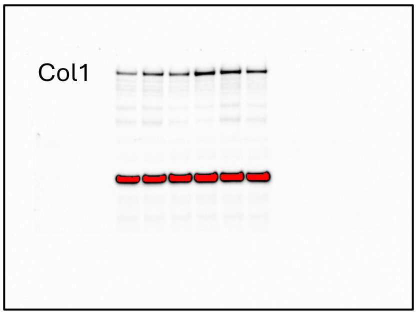

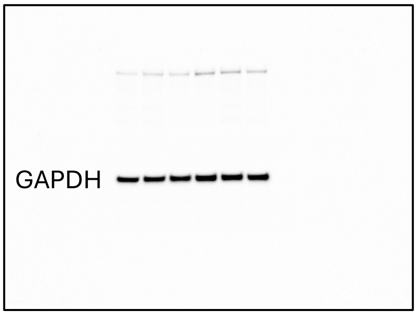

Figure S7B

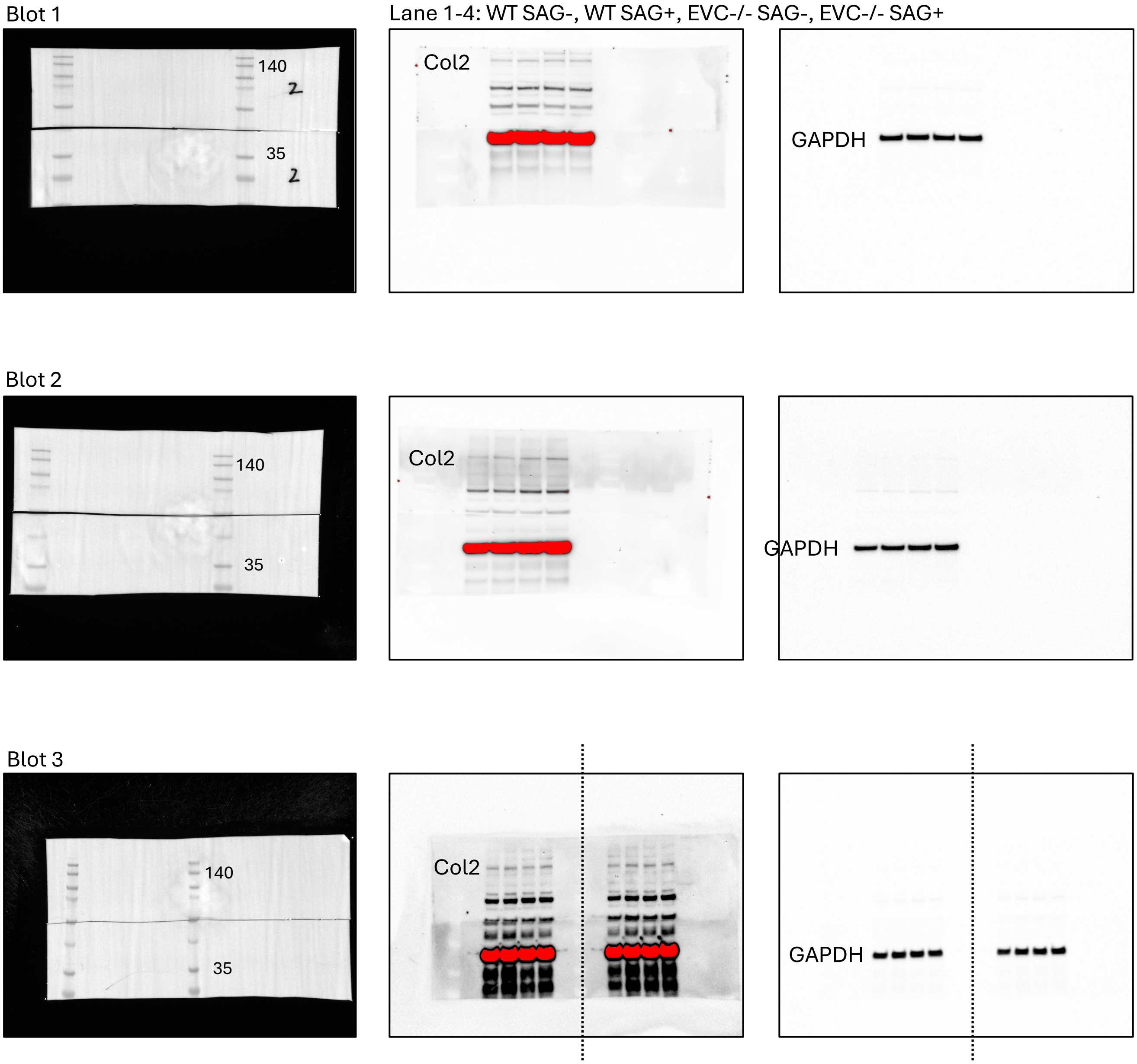
Blot 1
Lane 1-4: WT SAG-, WT SAG+, EVC-/- SAG-, EVC-/- SAG+
140
2
35
2
Col2
GAPDH
Blot 2
140
35
Col2
GAPDH
Blot 3
140
35
Col2
GAPDH
